# Histone modification-regulated LncRNA *DLEU1* interacts with ASCC2/ALKBH3 complex to drive DNA repair, antioxidant homeostasis and glucose metabolism in gastric cancer

**DOI:** 10.1186/s40364-025-00867-y

**Published:** 2026-01-02

**Authors:** Xiaoyan Zhang, Xin Wang, Qi Wang, Xu Wang, Hui Sun, Yingxue Liu, Cong Tan, Shujuan Ni, Weiwei Weng, Meng Zhang, Lei Wang, Dan Huang, Jie Chen, Xiaoyu Wang, Lu Gan, Mierxiati Abudurexiti, Wenfeng Wang, Jinjia Chang, Weiqi Sheng, Midie Xu

**Affiliations:** 1 Department of Pathology, Fudan University Shanghai Cancer Center, Shanghai, 200032 China; 2 Department of Oncology, Shanghai Medical College, Fudan University, Shanghai, 200032 China; 3 Institute of Pathology, Fudan University, Shanghai, 200032 China; 4 Department of Gastric Surgery, Fudan University Shanghai Cancer Center, Shanghai, 200032 China; 5 Laboratory of Immunology and Virology, Experiment Center for Science and Technology, Shanghai University of Traditional Chinese Medicine, Shanghai, 201203 China; 6 Department of Medical Oncology, Fudan University Zhongshan Hospital, Shanghai, 200032 China; 7 Department of Urology, Shanghai Pudong New Area Gongli Hospital, Shanghai, 200135 China; 8 Cancer Institute, Shanghai Urological Cancer Institute, Department of Oncology, Shanghai Medical College, Fudan University, Shanghai Cancer Center, Fudan University, Shanghai, 200032 China; 9 Department of Medical Oncology, Fudan University Shanghai Cancer Center, Shanghai, 200032 China

**Keywords:** DLEU1, Gastric cancer, DNA repair, Redox homeostasis, Glucose metabolism

## Abstract

**Background:**

Long non-coding RNA (lncRNA) *DLEU1* has been implicated in tumorigenesis, yet its mechanistic role in gastric cancer (GC) remains elusive.

**Methods:**

We investigated the epigenetic regulation and oncogenic function of *DLEU1* in GC through chromatin immunoprecipitation, RNA-protein interaction assays, and functional analyses in organoids and xenograft models. The molecular mechanisms underlying *DLEU1*-mediated DNA repair and metabolic adaptation were elucidated using western blotting, quantitative RT-PCR, and luciferase reporter assays.

**Results:**

*DLEU1* was significantly upregulated in GC, driven by H3K27 acetylation and H3K4 methylation. Mechanistically, *DLEU1* promoted DNA repair by facilitating ASCC2 nuclear translocation and its interaction with ALKBH3, thereby stabilizing *E2F1* mRNA. In turn, E2F1 directly activated *G6PD* transcription, leading to enhanced NADPH production, redox homeostasis, and glucose metabolism. Functionally, co-targeting *DLEU1* and ASCC2 synergized with G6PD inhibition, significantly impairing GC cells *via*bility and tumor growth.

**Conclusion:**

Our findings establish *DLEU1* as a key oncogenic lncRNA in GC, orchestrating DNA repair, redox balance, and metabolic adaptation *via* the ASCC2-ALKBH3-E2F1-G6PD axis. Targeting this pathway may provide a promising therapeutic strategy for overcome GC chemoresistance.

**Supplementary Information:**

The online version contains supplementary material available at 10.1186/s40364-025-00867-y.

## Highlights

DLEU1 expression is epigenetically activated in GC.

DLEU1-ASCC2-ALKBH3 axis enhances DNA repair and stabilizes E2F1.

E2F1 drives G6PD-mediated metabolic adaptation in GC.

Dual targeting of DLEU1/ASCC2 and G6PD exerts synergistic antitumor effects

## Introduction

Gastric cancer (GC) is a significant global health challenge, ranking fifth in incidence and fourth in mortality among cancers [1]. Current treatment strategies, including surgery, systemic chemotherapy, HER2-targeted therapy, immunotherapy, and radiotherapy, have enhanced patient outcomes and survival [2]. However, many patients are diagnosed at advanced stages with metastasis, significantly limiting therapeutic options and highlighting the need for deeper insights into the molecular mechanisms driving GC progression [3].

Long non-coding RNAs (lncRNAs) are transcripts longer than 200 nucleotides that do not encode proteins but exert crucial regulatory functions in diverse biological processes [4]. Unlike traditional protein-coding RNAs, lncRNAs modulate gene expression and cellular pathways through multiple mechanisms, including transcriptional regulation [5], RNA degradation [6], chromatin modification [7], nuclear-cytoplasmic transport [8]. Several oncogenic lncRNAs have been implicated in GC progression. For example, we previously reported that *nuclear-enriched abundant transcript 1 (NEAT1)* facilitates colorectal cancer progression by stabilizing the DEAD-box helicase 5 (DDX5) and activating Wnt/β-catenin signaling pathway [9]. Similarly, *plasmacytoma variant translocation 1 (PVT1)* promotes GC development by stabilizing forkhead box M1 (FOXM1), forming a positive feedback loop that further amplifies its own expression [10]. Given the pivotal role of lncRNA-protein interactions in tumorigenesis, a systematic investigation into their functional relevance in GC is warranted.

LncRNA *Deleted in Lymphatic Leukemia 1 (DLEU1)* has emerged as a key regulator in cancer progression. Previous studies suggest that *DLEU1* recruits epigenetic regulators, such as lysine-specific demethylase 1 (LSD1) [11], SET and MYND domain containing 2 (SMYD2) [12], enhancing its functional repertoire in GC. In this study, we demonstrate that *DLEU1* drives GC tumorigenesis by promoting DNA repair, metabolic adaptation, and cell proliferation. Mechanistically, *DLEU1* facilitates the nuclear translocation of activating signal cointegrator 1 complex subunit 2 (ASCC2), which subsequently interacts with the AlkB homolog 3 (ALKBH3). This interaction not only boosts DNA damage repair [13] but also stabilizes *E2F transcription factor 1 (E2F1)* mRNA. In turn, E2F1 activates the *glucose-6-phosphate dehydrogenease (G6PD)* transcription, driving NADPH production and metabolic reprogramming to support tumor growth and metastasis. Functionally, silencing *DLEU1* in combination with ASCC2 depletion and G6PD inhibition significantly impairs GC progression in vivo, underscoring the therapeutic potential of targeting the *DLEU1*/ASCC2/G6PD axis.

## Materials and methods

### Tissue collection and ethics statement

For the RT-qPCR detection of gene expression, we collected 203 cancer cases and 96 corresponding normal gastric tissue samples stored in liquid nitrogen from the Biobank of Fudan University Shanghai Cancer Center (FUSCC). A series of tissue microarrays (TMA) from the Biobank, which included 130 GC cases, were utilized for the fluorescent multiplex immunohistochemistry. None of the patients received specific treatment prior to surgery. All tissues included in the study were confirmed to be GC through histopathological evaluation. This study was approved by the Ethics Committee of FUSCC, and informed consent was obtained from each patient. The study was approved by the Research Ethics Committee, and all patients provided informed consent (Ethical code: 2407-ZZK-124).

### Fluorescent multiplex immunohistochemistry (mIHC), tissue imaging, and analysis

RNA in situ hybridization of *DLEU1* mRNA was performed using the multiplex fluorescence RNA in situ hybridization kit (Alpha X Bio, China, #AXC25024031). Cells were fixed with 10% NBF and incubated with preA solution at room temperature to inhibit endogenous peroxidase activity. After proteinase treatment, the samples were hybridized with probes at 40 °C for 2 h, followed by signal amplification. Finally, the target RNA was labeled with green fluorescence using tyramide signal amplification (TSA) and a tyramide substrate.

Formalin-fixed paraffin-embedded (FFPE) tissue Sects. (2–5 μm) were deparaffinized with xylene, rehydrated through a gradient of ethanol, and subjected to heat-induced epitope retrieval (HIER) using EDTA buffer. For mIHC, the AlphaXPainter X3 system was used with three panels. The primary antibodies were incubated at 37 °C for 1 h, followed by incubation with Alpha X poly HRP Ms + Rb conjugates for 10 min. Visualization was performed using the Alpha X 7-color IHC kit (Alpha X Bio, China, #AXT37100031). The slides were scanned using the ZEISS AXIOSCAN 7(Carl Zeiss AG, Germany).

The images from the two groups were separately overlaid and analyzed using HALO software (Indica Labs, USA). The staining sequence and dye combination are as follows: *DLEU1* probe (NR_109973.1, 5-1627, working solution, Alpha X Bio, China) - TSA520; E2F1 (1/200 dilution, Proteintech, Cat. #66515-1-Ig, RRID: AB_2881878) - TSA480; ALKBH3 (1/200 dilution, Proteintech, Cat. #12292-1-AP, RRID: AB_11125161) - TSA620; and ASCC2 (1/200 dilution, Proteintech, Cat.#11529-1-AP, RRID: AB_2059358) - TSA780.

### Cell lines and culture conditions

We purchased six GC cell lines (HGC27, AGS, MKN45, MGC803, NCI-N87, and SGC7901) and a healthy cell line (GES-1) from the Institute of Biochemistry and Cell Biology, Chinese Academy of Sciences (Shanghai, China). The cells were cultured in RPMI 1640 medium containing 10% fetal bovine serum, 100 U/ml penicillin, and 100 mg/ml streptomycin, under humidified conditions at 37 °C with 5% CO_2_.

### Organoid isolation and culture

Organoids were constructed in the Living Biobanks of FUSCC, which was developed in response to the pivotal applications of organoids in disease modeling and pharmaceutical field [14]. Fresh GC and normal tissues were collected, minced (1–3 mm³), and washed in PBS. After enzymatic digestion (trypsin, collagenase IV, DNase I) at 37 °C for 30–60 min, cell aggregates were filtered (70 μm), mixed with Matrigel (1:1, Corning, USA, #356234), and seeded in 24-well plates. After polymerization (37 °C, 15 min), organoid culture medium was added, and cultures were maintained at 37 °C, 5% CO₂. Organoids were harvested after 7–14 days for passaging and characterized by GC markers (CEA, 1/200 dilution, Proteintech, Cat. #68377-1-Ig, RRID: AB_3085095; CK7, 1/200 dilution, Beijing Zhongshan Golden Bridge Biotechnology, Cat. #ZM-0071, RRID: none). For viral transfection, organoids were incubated with viral suspension (4 °C, 1 h), infected (6–8 h), and resuspended in Matrigel for further culture. For drug assays, organoids in 96-well plates were treated with Etoposide (MCE, China, #HY-13629), and *via*bility was assessed using CellTiter-Glo 3D (Promega, USA, #G9681) to evaluate proliferation inhibition and apoptosis induction.

### Chromatin isolation by RNA purification (ChIRP)

ChIRP experiments were performed using the EZ-Magna ChIRP Kit (Millipore, Germany, #17-10495). Cells were subjected to formaldehyde crosslinking to stabilize RNA-protein-DNA complexes prior to lysis. The resultant lysate underwent hybridization with biotinylated probes tailored to target specific RNA sequences. To isolate the complexes, streptavidin-coated magnetic beads were employed, followed by rigorous washing to eliminate non-specific interactions. The captured complexes were subsequently eluted and analyzed through mass spectrometry after gel electrophoresis.

### mRNA stability assay

The mRNA stability assay was performed using the transcription inhibitor Actinomycin D (5 µg/mL, MCE, China, #HY-17559) to assess the effect of ALKBH3 on E2F1 mRNA stability. Cells were transfected with Vector or ALKBH3, followed by Actinomycin D treatment. RNA was extracted at different time points, and E2F1 mRNA levels were quantified using RT-qPCR.

### Cell Immunofluorescence

Initially, cells are cultured on coverslips and fixed using 4% paraformaldehyde at room temperature for 15 min. Following fixation, permeabilization is achieved with 0.1% Triton X-100 for 10 min, and non-specific binding is minimized through a one-hour incubation with 5% bovine serum albumin (BSA). The primary antibodies, ASCC2 (1:200, Proteintech, Cat. #11529-1-AP, RRID: AB_2059358) and γH2AX (1:200, Huaan Biosciences, Cat.#ET1602-2, RRID: AB_3069632), are then applied and incubated overnight at 4 °C. After washing the cells, fluorophore-conjugated secondary antibodies are introduced at room temperature for one hour, with nuclei stained using DAPI for 5 min. Finally, samples are mounted with anti-fade medium and examined under a fluorescence microscope, allowing for the capture of images essential for analyzing protein localization and expression.

### Luciferase reporter assay

Luciferase reporter assay were performed using the Dual-Luciferase^®^ Reporter Assay System (Promega, USA, #E1910) according to the manufacturer’s instructions. Cells were co-transfected with firefly luciferase reporter plasmid and a Renilla luciferase plasmid. After 24–48 h, luciferase activity was measured using a microplate luminometer. Firefly luciferase activity was normalized to Renilla luciferase activity to control for transfection efficiency.

### NADPH levels and NADPH/NADP⁺ ratio measurement

NADPH levels and the NADPH/NADP⁺ ratio were measured using the NADP/NADPH Quantification Kit (Beyotime, Cat. #S0180S) following the manufacturer’s protocol. Briefly, cells were lysed with NADP/NADPH extraction buffer, and the supernatants were collected after centrifugation at 12,000 × g for 10 min. NADP⁺ and NADPH were quantified by enzymatic cycling reactions, and the absorbance was measured at 450 nm using a microplate reader.

### ROS level detection

Reactive oxygen species (ROS) levels were detected using the DCFH-DA probe (Beyotime, Cat. #S0033S). Cells were incubated with 10 µM DCFH-DA at 37 °C for 20 min in the dark. After washing with PBS, intracellular ROS levels were measured using flow cytometry.

### EdU incorporation assay

DNA synthesis was evaluated using the EdU (5-ethynyl-2’-deoxyuridine) incorporation assay with the EdU Assay Kit (Beyotime, Cat. #C0075S). Cells were incubated with 10 µM EdU for 2 h, fixed with 4% paraformaldehyde, and permeabilized with 0.5% Triton X-100. EdU staining was performed using Alexa Fluor 555-conjugated azide according to the kit instructions, followed by nuclear staining with Hoechst. Finally, the cells were observed under an inverted fluorescence microscope.

### G6PD enzymatic activity assay

Glucose-6-phosphate dehydrogenase activity was measured using the G6PD Activity Assay Kit (Beyotime, Cat. #S0189). Cell lysates were prepared according to the manufacturer’s protocol, and enzymatic activity was assessed by monitoring the reduction of NADP⁺ to NADPH at 450 nm over time using a microplate reader.

### In vivo experiments

Male nude mice (5 weeks old) were maintained in pathogen-free conditions. Animals were assigned to experimental groups using simple randomization. We injected 1 × 10^7 cells/ml of cancer cell lines (MKN45) and their stable transfectants with relevant genes subcutaneously into the flanks of the mice. Starting from day 7, the length (L) and width (W) of the tumors were recorded. Tumor volume was calculated using the formula: V = (L×W²)/2. For treatment, it started two weeks post-tumor formation. Etoposide (MCE, China, #HY-13629 was administered at a dose of 10 mg/kg per mouse, every three days for a total of five doses. G6PDi-1 (MCE, #HY-W107464) was administered at 10 mg/kg per mouse, every two days for a total of six doses. After two weeks, all mice were euthanized, and the tumors were surgically removed and measured. All animal procedures were approved by the Ethics Committee of the Experimental Animal Center at our hospital, with the ethical approval code: FUSCC-IACUC-S2022-0358.

Other methods used in this study were described in previous publications and are listed in the Supplementary Information [10, 15, 16].

### Statistical analysis

Statistical analyses and data visualization were conducted using GraphPad Prism 10.1.2 and R Studio 4.2.3. For comparisons between two groups, the Shapiro-Wilk test was first performed to assess normality and homogeneity of variance. If these assumptions were met, a Student’s t-test was used; otherwise, the Mann-Whitney U test was applied. For multiple-group comparisons, one-way ANOVA was performed if normality and homogeneity of variance were satisfied; otherwise, the Kruskal-Wallis test was used. For continuous variables, *Pearson* correlation analysis was conducted if the data followed a normal distribution and exhibited a linear correlation; otherwise, *Spearman* correlation analysis was applied. All data are presented as mean ± SEM, with experiments performed in triplicate. Statistical significance was defined as *P<0.05, **P<0.01, ***P<0.001, while “ns” indicates no statistically significant difference.

## Results

### Histone modification-activated LncRNA *DLEU1* is upregulated in human GC tissues and correlates with poor clinical prognosis

**Fig. 1:**
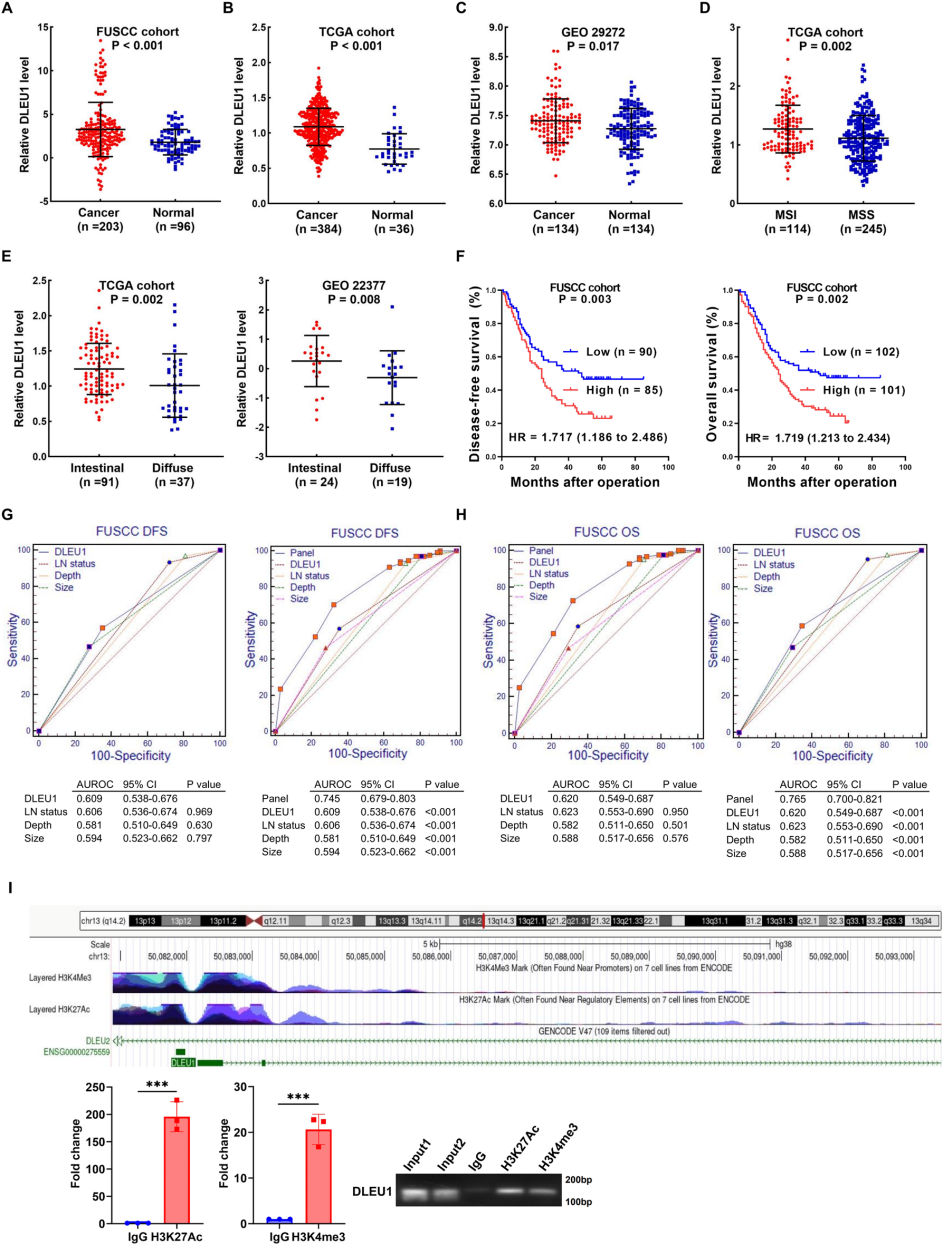
*DLEU1* is upregulated in gastric cancer and is associated with poor prognosis. **A**–**C**. Relative expression of *DLEU1* in GC tissues compared to normal tissues in the FUSCC cohort (**A**), TCGA cohort (**B**), and GEO dataset GSE29272 (**C**). **D**. Comparison of *DLEU1* expression between microsatellite instability (MSI) and microsatellite stability (MSS) subtypes in the TCGA cohort. **E**. Relative *DLEU1* expression in intestinal and diffuse subtypes of GC in the TCGA cohort (left) and GSE22377 dataset (right). **F**. Kaplan-Meier survival curves for disease-free survival (DFS, left) and overall survival (OS, right) in the FUSCC cohort. **G**–**H**. Receiver operating characteristic (ROC) curve analyses evaluating the prognostic performance of *DLEU1*, depth of invasion (Depth), Lymphatic metastasis (LM), and TNM stage for DFS (**G**) and OS (**H**) in the FUSCC cohort. **I**. Genomic landscape of the *DLEU1* locus highlighting H3K27Ac and H3K4me3 peaks, indicative of active regulatory elements (upper). Chromatin immunoprecipitation (ChIP) assays confirmed the enrichment of these histone modifications at the *DLEU1* promoter (lower). *, *P* < 0.05; **, *P* < 0.01; ***, *P* < 0.001; ns, not significant

We investigated the expression and clinical significance of *DLEU1* in GC by analyzing its mRNA levels in tumor and adjacent non-cancerous tissues from patients in the FUSCC cohort. Our results revealed a significant upregulation of *DLEU1* in GC samples compared to normal counterparts (*P* < 0.001; Fig. 1A). This observation was further supported by independent analyses of The Cancer Genome Atlas (TCGA) and the GEO29272 datasets (Fig. 1B-C). Notably, *DLEU1* expression was markedly higher in patients with high microsatellite instability (MSI-H) [17, 18] compared to those with microsatellite stability (MSS) in the TCGA cohort (*P* = 0.002; Fig. 1D). Moreover, patients with intestinal-type GC exhibited markedly elevated *DLEU1* levels compared to those with diffuse-type subtype [19], a finding consistently observed in both the TCGA (*P* = 0.002) and GEO22377 cohorts (*P* = 0.008, Fig. 1E).

Clinically, elevated *DLEU1* expression was significantly associated with increased tumor infiltration depth (Table 1, *P* = 0.040). Additionally, patients with higher *DLEU1* levels exhibited significantly poorer disease-free survival (DFS; *P* = 0.003) and overall survival (OS; *P* = 0.002) compared to those with lower expression (Fig. 1F). Univariate and multivariate Cox proportional hazards analyses (Tables 2 and 3) identified *DLEU1*, along with tumor size, infiltration depth, and lymphatic metastasis (LM), as independent prognostic factors for DFS (Fig. 1G) and OS (Fig. 1H). Although receiver operating characteristic (ROC) curves indicated that *DLEU1* alone did not outperform other prognostic factors in predicting DFS and OS, its combination with LM, tumor depth, and size provided enhanced prognostic accuracy (Fig. 1G-H). Collectively, these findings underscore *DLEU1* as a valuable prognostic biomarker in GC.

**Table 1:** Relationship between *DLEU1* expression and clinicopathologic parameters of gastric cancer patients

Characteristics	Number of case	DLEU1 expression				*P* value
		Low (*n* = 102)	%	High (*n* = 101)	%	
Age (years)	203	60.04 ± 10.73		60.92 ± 9.75		0.159
Gender						0.626
Male	154	79	77.5	75	74.3	
Female	49	23	22.5	26	25.7	
Tumor size						0.569
< 5 cm	121	63	61.8	58	57.4	
≥ 5 cm	82	39	38.2	43	42.6	
Location						0.424
Upper stomach	48	20	19.6	28	27.7	
Middle stomach	67	38	37.3	29	28.7	
Lower stomach	68	35	34.3	33	32.7	
Entire stomach	20	9	8.8	11	10.9	
Histologic grade						0.422
Well/moderately	51	23	22.5	28	27.7	
Poorly/others	152	79	77.5	73	72.3	
Depth of invasion						0.040*
T1,T2	17	13	12.7	4	4	
T3,T4	186	89	87.3	97	96	
Lymphatic metastasis						0.839
Absent	28	15	14.7	13	12.9	
Present	175	87	85.3	88	87.1	
Vascular invasion						0.15
Absent	79	45	44.1	34	33.7	
Present	124	57	55.9	67	66.3	
Nervous invasion						0.467
Absent	74	40	39.2	34	33.7	
Present	129	62	60.8	67	66.3	
Distant metastasis						0.423
Absent	175	90	88.2	1385	84.2	
Present	28	12	11.8	16	15.8	
TNM stage						0.294
I and II	41	24	23.5	17	16.8	
III and IV	162	78	76.5	84	83.2	

**Table 2:** Univariate and multivariate analysis of clinicopathological factors for disease-free survival in gastric cancer

Variable	Univariate analysis		Multivariate analysis	
	HR (95% CI)	p^a^	HR (95% CI)	p^a^
Age	1.138 (0.809–1.603)	0.458		
(< 60/≥60)				
Gender	1.248 (0.852–1.828)	0.256		
(Male/Female)				
Location	1.147 (0.955–1.378)	0.143		
(Upper/middle/lower/Entire)				
Tumor size	1.579 (1.125–2.216)	0.008*	1.746 (1.239–2.460)	0.001*
(≥ 5/<5)				
Histologic grade	1.554 (1.030–2.346)	0.036*		
(Well, mod/Poor, others)				
Depth of tumor	4.489 (1.658–12.158)	0.003*	2.891 (1.057–7.907)	0.039*
(T3, T4/T1, T2)				
Vascular invasion	1.750 (1.214–2.523)	0.003*		
(Present/Absent)				
Nervous invasion	1.861 (1.280–2.704)	0.001*		
(Present/Absent)				
Lymphatic metastasis	3.376 (1.713–6.654)	0.000*	3.248 (1.629–6.473)	0.001*
(Present/Absent)				
Distant metastasis	1.727 (1.102–2.708)	0.017*		
(Present/Absent)				
TNM stage	3.215 (1.844–5.607)	0.000*		
(III + IV/I + II)				
*DLEU1*	1.635 (1.160–2.302)	0.005*	1.571 (1.113–2.218)	0.010*
(High /Low)				

HR Hazard ratio, CI confidence interval, ^a^ All statistical tests were 2-sided. Significance level: *P* < 0.05

**Table 3:** Univariate and multivariate analysis of clinicopathological factors for overall survival in gastric cancer

Variable	Univariate analysis		Multivariate analysis	
	HR (95% CI)	*p* ^a^	HR (95% CI)	*p* ^a^
Age (< 60/≥60)	1.242 (0.873–1.766)	0.229		
Gender (Male/Female)	1.279 (0.867–1.887)	0.215		
Location (Upper/middle/lower/Entire)	1.165 (0.966–1.405)	0.109		
Tumor size (≥ 5/<5)	1.569 (1.108–2.222)	0.011*	1.786 (1.255–2.541)	0.001*
Histologic grade (Well, mod/Poor, others)	1.651 (1.073–2.542)	0.023*		
Depth of tumor (T3,T4/T1,T2)	4.436 (1.728–17.104)	0.004*	3.256 (1.026–10.331)	0.045*
Vascular invasion (Present/Absent)	1.767 (1.212–2.578)	0.003*		
Nervous invasion (Present/Absent)	1.975 (1.336–2.918)	0.001*		
Lymphatic metastasis (Present/Absent)	4.735 (2.083–10.761)	0.000*	4.532 (1.974–10.402)	0.000*
Distant metastasis (Present/Absent)	1.646 (1.039–2.607)	0.034*		
TNM stage (III + IV/I + II)	3.912 (2.103–7.277)	0.000*		
*DLEU1* (High /Low)	1.728 (1.214–2.459)	0.002*	1.679 (1.177–2.397)	0.004*

HR Hazard ratio, CI confidence interval, ^a^ All statistical tests were 2-sided. Significance level: *P* < 0.05

To investigate the mechanisms underlying the aberrant upregulation of *DLEU1* in GC, we examined its promoter landscape using the UCSC Genome Browser (http://genome.ucsc.edu/) [20]. This analysis revealed significant enrichment of H3K27Ac and H3K4me3, histone marks associated with active transcription, at the *DLEU1* promoter. ChIP assays further demonstrated an increased deposition of H3K27Ac and H3K4me3 at this locus in HGC27 GC cells (Fig. 1I). Collectively, these findings support the notion that histone modifications contribute to the transcriptional activation of *DLEU1* in GC, providing mechanistic insight into its dysregulated expression.

### *DLEU1* attenuates DNA damage accumulation and promotes glycolysis in GC cells

**Fig. 2:**
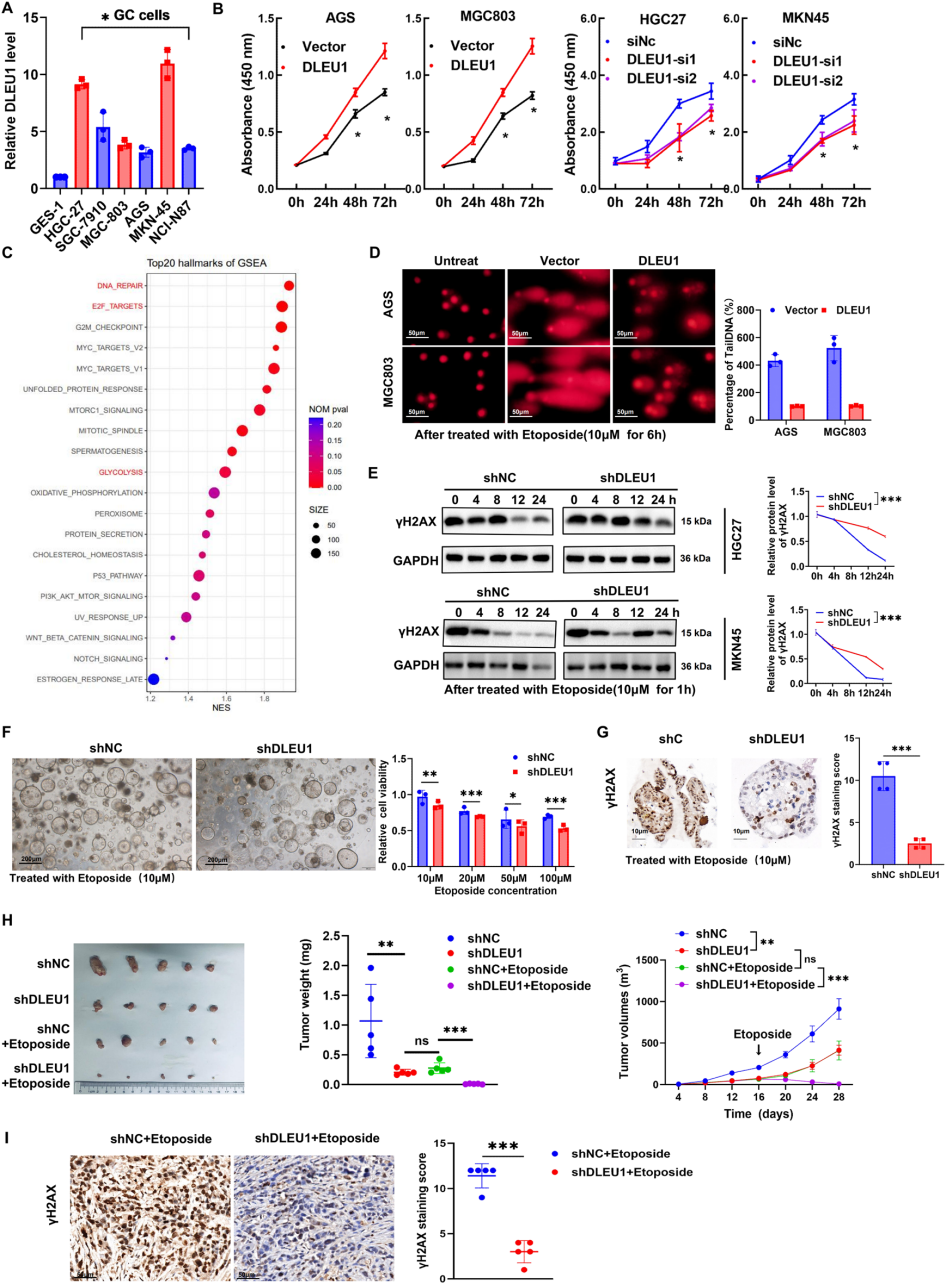
ADLEU1 promotes GC cells proliferation, DNA damage repair, and metabolic reprogramming. **A**. Relative DLEU1 expression levels in gastric cancer (GC) cell lines (AGS, MGC803, HGC27, MKN45) compared to a normal gastric epithelial cell line (GES-1). **B**. Cell proliferation assays in GC cell lines transfected with DLEU1 overexpression plasmid, empty vector, siRNAs targeting DLEU1, or control siRNA (siNC). **C**. Gene set enrichment analysis (GSEA) showing significant enrichment of DNA repair, metabolic pathways and E2F1 targets upon DLEU1 overexpression. **D**. Comet assay results demonstrating reduced DNA damage in DLEU1-overexpressing AGS and MGC803 cells, compared to the empty vector transfected group. Representative images and quantification of tail moments are shown. Scale bar =20μm. **E**. Western blot analyzed of γH2AX levels in HGC27 and MKN45 cells transfected with DLEU1 shRNA (shDLEU1) or control shRNA (shNC), following Etoposide treatment (10 µM for 1 hour). **F**. Cell viability analysis of GC organoid transfected with shDLEU1 or shNC, after treated with increasing concentrations of Etoposide. Left panel: Quantitative viability analysis. Right panel: Representative images of tumor organoids treated with 10 µM Etoposide. Scale bar =200μm. **G**. Representative immunohistochemistry (IHC) images showing γH2AX expression in GC organoid transfected with shDLEU1 or shNC, after treated with Etoposide (10 μM). Scale bar = 10 μm. **H**. Etoposide treatment synergistically inhibited tumor growth in DLEU1-knockdown xenograft models. Left panel: Representative tumor images from shNC, shDLEU1, shNC+Etoposide, and shDLEU1+Etoposide groups. Middle panel: Tumor weight analysis across different groups. Right panel: Tumor volume progression over time. **I**. γH2AX staining revealed increased DNA damage upon Etoposide treatment in DLEU1-knockdown tumors. Left panel: Representative IHC staining for γH2AX in shNC+Etoposide and shDLEU1+Etoposide groups. Scale bar =50μm. Right panel: Quantification of γH2AX staining scores. *, P < 0.05; **, P < 0.01; ***, P < 0.001; ns, not significant. See also Figures S1 and S2.

To elucidate the functional role of *DLEU1* in GC, we analyzed its endogenous expression across a panel of GC cells. RT-qPCR analysis revealed markedly elevated *DLEU1* expression in GC cells compared with normal human gastric epithelial cells GES-1 (Fig. 2A). To further assess its functional implications, we modulated its expression by overexpressing *DLEU1* in MGC803 and AGS cells while silencing it in HGC27 and MKN45 cells (Supplementary Fig. 1A). Overexpression of *DLEU1* significantly enhanced proliferation and colony-forming ability, whereas its knockdown significantly impaired both (Fig. 2B, Supplementary Fig. 1B). Additionally, *DLEU1* overexpression promoted cell migration and invasion, while its depletion suppressed these metastatic properties (Supplementary Fig. 1C-D).

Given the observed correlation between *DLEU1* overexpression and MSI-H status in GC (Fig. 1D), we hypothesized that *DLEU1* may regulate DNA damage repair. RNA sequencing and gene enrichment analyses identified a significant upregulation of DNA repair-related genes in *DLEU1*-overexpressing AGS cells (Fig. 2C). Subsequent comet assay analyses confirmed that *DLEU1* overexpression reduced DNA damage, as indicated by diminished comet tail parameters, whereas *DLEU1* knockdown led to increased DNA fragmentation (Fig. 2D, Supplementary Fig. 1F). Furthermore, following DNA damage induction, *DLEU1*-overexpressing cells exhibited accelerated γ-H2AX clearance (a biomarker of DNA damage [21]), suggesting enhanced DNA repair efficiency, while *DLEU1* depletion prolonged γ-H2AX persistence (Fig. 2E, Supplementary Fig. 1G). Consistent with these findings, apoptosis assays also indicated that *DLEU1* overexpression reduced apoptotic responses, whereas its knockdown heightened apoptosis (Supplementary Fig. 1H). To determine whether *DLEU1* regulates the expression of key mismatch repair (MMR) genes, we performed RT–qPCR analyses in AGS and MGC803 gastric cancer cell lines following *DLEU1* overexpression. The results revealed a marked upregulation of *MLH1*, *MSH6*, and *PMS2* mRNA levels, whereas MSH2 expression remained largely unchanged (Supplementary Fig. 1I). These findings suggest that *DLEU1* may enhance DNA mismatch repair activity, thereby promoting DNA repair capacity and supporting the survival of gastric cancer cells.

Beyond its role in genome stability, *DLEU1* also modulates cellular metabolism. Gene set enrichment analysis (GSEA) revealed a significant enrichment of glycolysis and oxidative phosphorylation pathways in *DLEU1*-overexpressing cells (Fig. 2C). Functional assays demonstrated that *DLEU1* overexpression enhanced glucose uptake, lactate production, and ATP generation, while knockdown of *DLEU1* inhibited these metabolic processes (Supplementary Fig. 2A). Furthermore, extracellular acidification rate (ECAR) and oxygen consumption rate (OCR) assays confirmed that *DLEU1* overexpression augmented both glycolytic flux activity and mitochondrial respiration (Supplementary Fig. 2B). Mechanistically, *DLEU1* overexpression upregulated key glycolytic regulators, including *GLUT1*, *GLUT4*, *HK2*, and *LDHA* (Supplementary Fig. 2C), suggesting a pivotal role for *DLEU1* in metabolic reprogramming in GC.

Given the strong association between *DLEU1* overexpression and MSI-H status in GC, as well as RNA sequencing data indicating enrichment of DNA damage repair pathways (Figs. 1D and 2C), we investigated whether *DLEU1* modulated chemotherapy response. Using GC organoid models [22], 3D *via*bility assays showed that *DLEU1* knockdown significantly reduced organoid *via*bility following Etoposide treatment, suggesting heightened chemosensitivity. Consistently, γH2AX IHC staining revealed a diminished DNA damage response in *shDLEU1* organoids (Fig. 2F-G, Supplementary Fig. 2D). Similarly, in a mouse xenograft model, *DLEU1* knockdown synergistically enhanced Etoposide-induced tumor suppression, leading to a significantly greater reduction in tumor volume and weight compared to the *shNC* group (Figs. 2H). Furthermore, γH2AX staining demonstrated that tumors from the *DLEU1*-knockdown combined Etoposide treatment group exhibited lower expression of DNA damage repair markers, further supporting a role for *DLEU1* in DNA damage resolution (Fig. 2I).

Taken together, these findings demonstrate that *DLEU1* is highly expressed in GC cells and drives malignant phenotypes by promoting cell proliferation, migration, and metabolic metabolism, while also enhancing DNA damage repair and conferring chemoresistance may thus represent a promising therapeutic strategy for overcoming DNA repair–mediated drug resistance in GC.

### *DLEU1* promotes the nuclear localization of ASCC2 and facilitates ASCC2–ALKBH3 interaction

**Fig. 3:**
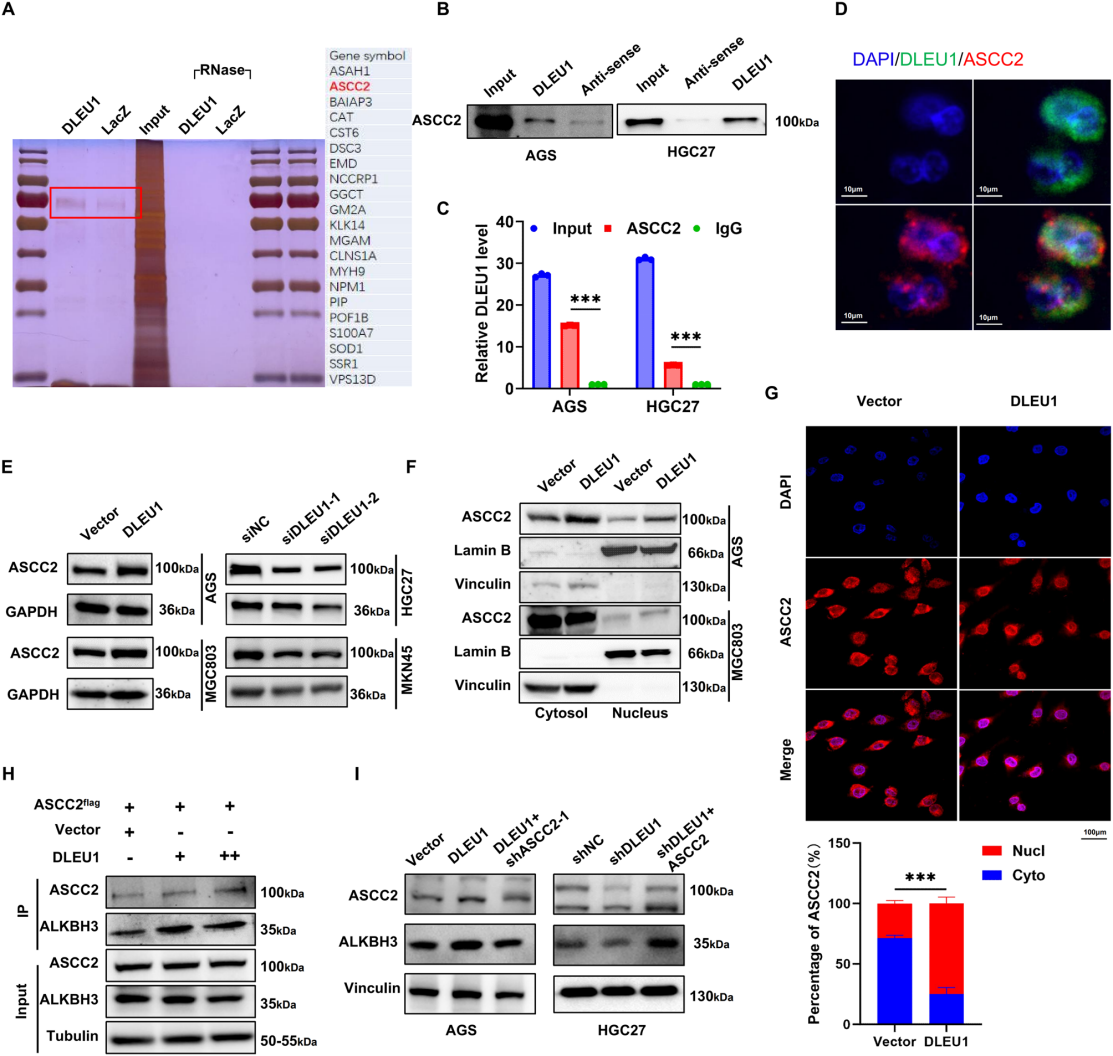
*DLEU1* facilitates ASCC2 nuclear translocation and promotes ASCC2–ALKBH3 interaction. **A**. Silver staining (left panel) analysis of proteins isolated from AGS cells using ChIRP with *DLEU1* or the control LacZ probes. The red rectangle highlighted the potential presence of ASCC2 in the *DLEU1* complex, subsequently confirmed by LC/MS mass spectrometry analysis (right panel). **B**-**C**. RNA pull-down (**B**) and RIP assays (**C**) demonstrated the interaction between ASCC2 and *DLEU1* in AGS and HGC27 cells. **D**. Immunofluorescence staining showed co-localization of *DLEU1* with ASCC2, Scale bar = 10 μm. **E**. Western blot results showed that overexpression of *DLEU1* increased ASCC2 protein levels in MGC803 and AGS cells, whereas knockdown of *DLEU1* decreased ASCC2 protein levels in HGC27 and MKN45 cells. **F**-**G**. Nuclear-cytoplasmic fractions (**F**) and Immunofluorescence staining (**G**) demonstrate increased nuclear localization of ASCC2 in *DLEU1* overexpressed cells. Scale bar = 10 μm. **H**. Endogenous co-immunoprecipitation assays reveal enhanced ASCC2–ALKBH3 binding following *DLEU1* overexpression. **I**. Western blot analysis showed changes in ASCC2 and ALKBH3 protein expression in AGS with *DLEU1* overexpression or combined *DLEU1* overexpression with ASCC2 knockdown(left panel), and in HGC27 cells with *DLEU1* knockdown or combined *DLEU1* knockdown with ASCC2 overexpression (right panel). *, *P* < 0.05; **, *P* < 0.01; ***, *P* < 0.001; ns, not significant. See also Figures S3

lncRNAs regulate gene expression through interactions with proteins in various cellular contexts [23]. To investigate protein interactions with *DLEU1* in a chromatin-associated environment while minimizing nonspecific binding, we performed chromatin isolation by RNA purification (ChIRP) using *DLEU1*-specific RNA probes. The purified complexes were analyzed *via* SDS-PAGE and visualized by silver staining, followed by mass spectrometry analysis (Fig. 3A, left). Liquid chromatography-mass spectrometry (LC/MS) analysis identified ASCC2 as a prominent *DLEU1*-associated protein (Fig. 3A, right). This interaction was further validated using biotin-RNA-protein pull-down assays coupled with Western blotting (Fig. 3B). Additionally, RIP assays validated that ASCC2 antibodies significantly enriched *DLEU1* in AGS and HGC27 cells (Fig. 3C). Immunofluorescence staining results revealed that *DLEU1* co-localized with ASCC2 within the cells (Fig. 3D). Collectively, these results indicate that *DLEU1* directly interacts with ASCC2.

To determine whether *DLEU1* regulates ASCC2 expression, we assessed its impact on *ASCC2* RNA and protein levels. RT-qPCR analysis showed that neither *DLEU1* overexpression nor knockdown significantly affected ASCC2 mRNA levels (Supplementary Fig. 3A). However, overexpression of *DLEU1* slightly increased ASCC2 protein levels, while silencing of *DLEU1* slightly reduced ASCC2 protein levels (Fig. 3E). Given that *DLEU1* did not alter ASCC2 mRNA expression, we examined whether it influenced ASCC2 protein stability using cycloheximide (CHX) chase assays. No significant changes in ASCC2 protein degradation were observed (Supplementary Fig. 3B). Next, we investigated the effect of *DLEU1* on ASCC2 intracellular localization. Nuclear-cytoplasmic fractionation revealed that *DLEU1* upregulation significantly increased the nuclear localization of ASCC2 compared to the control group (Fig. 3F). Immunofluorescence assays further confirmed that ASCC2 predominantly accumulated in the nucleus upon *DLEU1* overexpression (Fig. 3G).

ASCC2 is a ubiquitin-binding protein involved in DNA repair [24, 25] and ribosome quality control [26, 27]. It has been reported to recruit ALKBH3 to DNA damage sites by binding to polyubiquitinated proteins with “Lys-63” linkages [24]. To examine whether *DLEU1* modulated ASCC2–ALKBH3 interaction, we performed endogenous co-immunoprecipitation assays. Notably, overexpression of *DLEU1* enhanced ASCC2–ALKBH3 binding (Fig. 3H). Moreover, overexpression of *DLEU1* increased ALKBH3 protein levels, while silencing of ASCC2 partially abrogated this effect (Fig. 3I). Our data suggest that *DLEU1* exerts its function by binding to ASCC2, promoting its nuclear translocation, and facilitating its interaction with ALKBH3.

### ALKBH3 stabilize *E2F1* mRNA, facilitating its transcriptional activation of G6PD

**Fig. 4:**
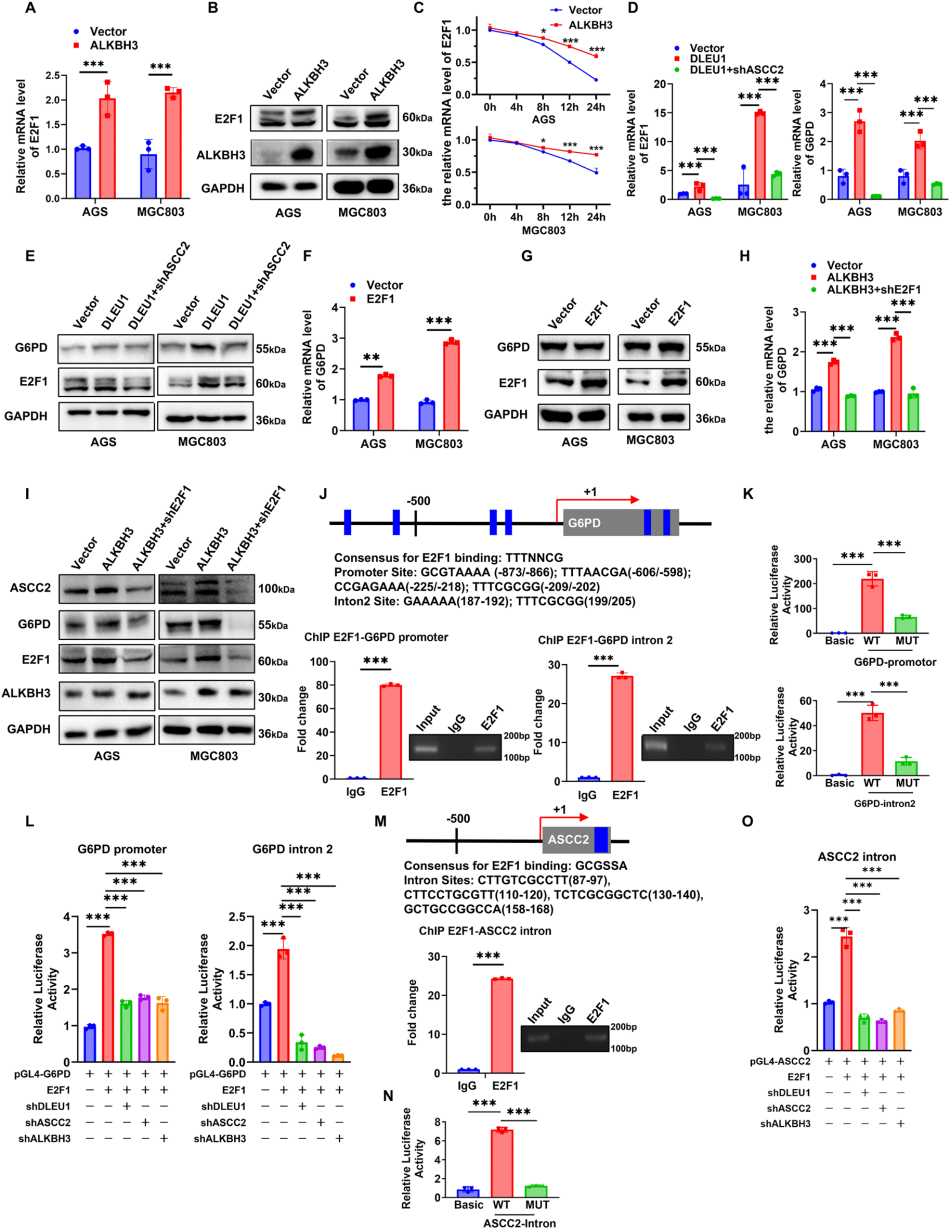
*DLEU1* promotes G6PD transcription *via* ASCC2/ALKBH3-mediated recruitment of E2F1. **A**-**B**. qRT-PCR and Western blot analysis of E2F1 expression in AGS and MGC803 cells upon ALKBH3 overexpression. **C**. Time-course analysis of E2F1 mRNA levels in AGS and MGC803 cells transfected with vector or ALKBH3. **D**-**E**. RT-qPCR and western blot analyzed of E2F1 and G6PD expression in AGS and MGC803 cells following *DLEU1* overexpression or *DLEU1* overexpression combined with ASCC2 knockdown. **F**-**G**. RT-qPCR and western blot analyzed of G6PD levels in AGS and MGC803 cells with E2F1 overexpression. **H**. qRT-PCR analysis of G6PD mRNA expression in AGS and MGC803 cells after ALKBH3 overexpression or co-transfection with ALKBH3 and shRNA targeting E2F1 (shE2F1). **I**. Western blot analysis of ASCC2, G6PD and E2F1 expression in AGS and MGC803 cells with ALKBH3 overexpression or ALKBH3 overexpression combined with E2F1 knockdown. **J**. ChIP-qPCR analysis confirmed E2F1 binding to the G6PD promoter and intron 2 regions in AGS cells. **K**. Luciferase reporter assay showed the transcriptional activity of wild-type and mutant G6PD promoter and intron 2 regions in AGS cells. **L**. Luciferase assays demonstrate that knockdown of *DLEU1*, ASCC2, or ALKBH3 attenuates E2F1-mediated activation of G6PD transcription. **L**-**M**. ChIP-qPCR and luciferase reporter validated E2F1 binding to an intronic region of ASCC2 in AGS cells. **M**. Luciferase assays indicate that knockdown of *DLEU1*, ASCC2, or ALKBH3 reduces E2F1-driven ASCC2 transcription. *, *P* < 0.05; **, *P* < 0.01; ***, *P* < 0.001; ns, not significant

ALKBH3 is the sole eraser of N1-methyladenosine (m¹A) in mRNA [28, 29]. Previous studies have reported that ALKBH3 enhances the stability of *E2F1* mRNA in an m1A demethylase activity dependent manner, thereby promoting E2F1-mediated transcriptional activation of key glycolytic enzymes [30]. Coincidentally, differentially upregulated genes in AGS cells overexpressing *DLEU1* were significantly enriched in E2F1 targets genes (Fig. 2C). Consistently, both *E2F1* mRNA and protein levels were upregulated in AGS and MGC803 cells overexpressing ALKBH3 (Fig. 4A-B). Additionally, we also confirmed that ALKBH3 enhanced the stability of *E2F1* mRNA (Fig. 4C). Transcriptomic analysis further revealed that among glycolytic enzymes, G6PD, a rate-limiting enzyme in the pentose phosphate pathway (PPP) [31], was the most significantly upregulated in *DLEU1*-overexpressing GC cells (Supplementary Table 1). GSEA also revealed significant enrichment of the glycolysis pathway, suggesting a coordinated upregulation of glucose metabolism. To delineate the mechanistic axis involving *DLEU1*, ASCC2, E2F1, and G6PD, we found that overexpression of *DLEU1* increased both E2F1 and G6PD at mRNA and protein levels, while silencing of ASCC2 partly attenuated this effect (Fig. 4D-E).

Functionally, overexpression of E2F1 increased both G6PD mRNA (Fig. 4F) and protein levels (Fig. 4G) while E2F1 knockdown abrogated ALKBH3-induced upregulation of G6PD (Fig. 4H-I). Furthermore, ChIP analysis confirmed direct E2F1 binding at two sites within the G6PD promoter and intron 2 regions (Fig. 4J), with luciferase reporter assays demonstrating that mutating these sites significantly reduced transcriptional activity (Fig. 4K). Additionally, knockdown of *DLEU1*, ASCC2 or ALKBH3 impaired E2F1-mediated transcriptional activation of G6PD (Fig. 4L). Interestingly, ChIP-seq analysis of E2F1 revealed strong enrichment at an intronic region of the ASCC2 (Supplementary Table 2). This finding was further validated by ChIP-qPCR experiments (Fig. 4M). Consistent with this, luciferase assays confirmed that mutations in binding sites significantly diminished luciferase activity (Fig. 4N). In addition, ASCC2 expression was also regulated by the ALKBH3/E2F1 axis (Fig. 4I and O), suggesting that E2F1 may regulate ASCC2 expression *via* intronic binding.

### *DLEU1* promotes DNA damage repair in GC *via* an ASCC2-dependent way

**Fig. 5:**
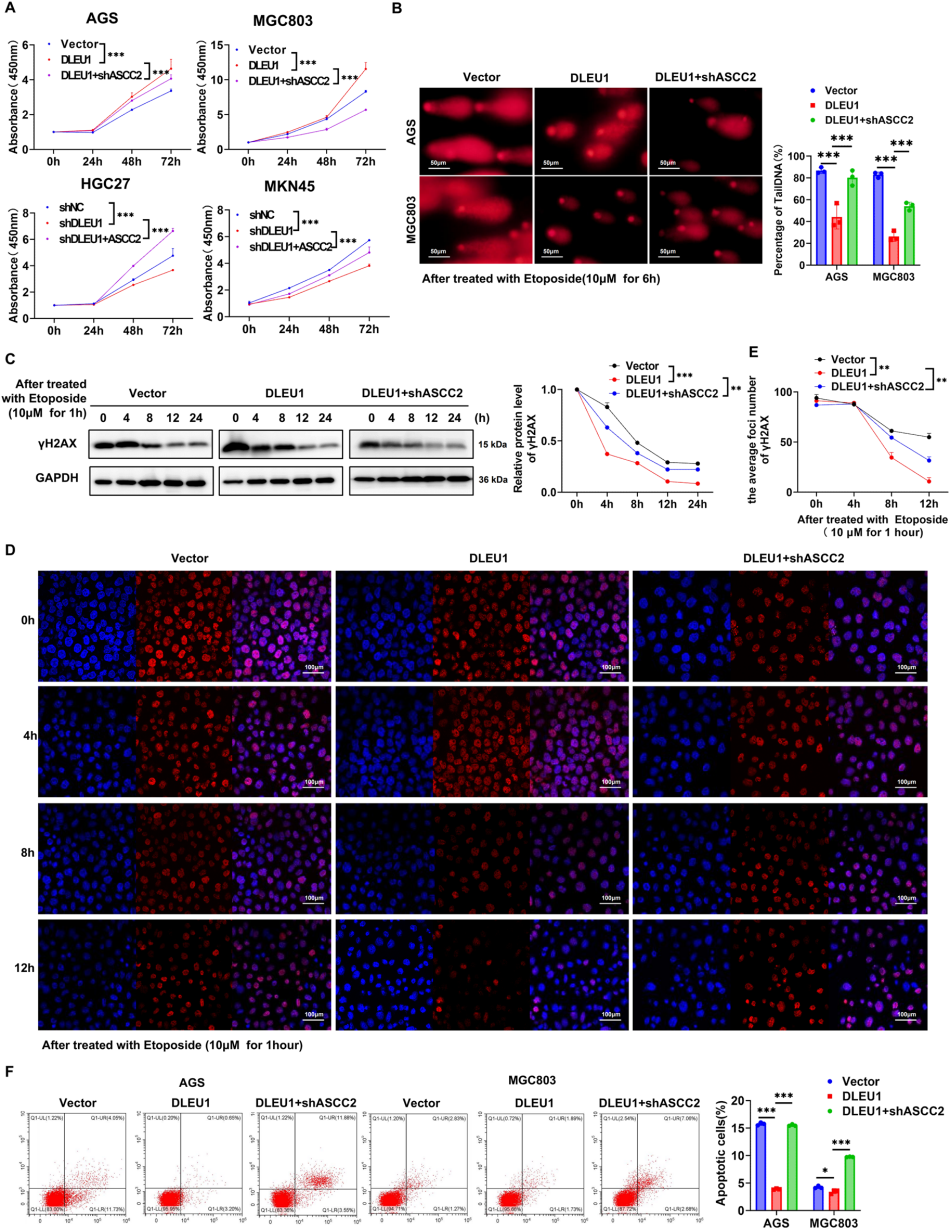
Inhibition of ASCC2 abrogates *DLEU1*-mediated DNA damage repair and apoptosis resistance in GC cells. **A**. Proliferation assays in AGS and MGC803 with *DLEU1* overexpression, with or without ASCC2 knockdown, and in HGC27 and MKN45 cells with *DLEU1* knockdown, with or without ASCC2 overexpression. **B**-**C**. Comet assay, Western blot analysis of γ-H2AX protein with *DLEU1* overexpression or *DLEU1* overexpression combined with ASCC2 knockdown. Scale bar = 50 μm. **D**-**E**. Immunofluorescence staining of γH2AX in AGS cells treated with Etoposide. Representative images at 0, 4, 8, and 12 h after treatment are shown. Quantification of average foci number is shown in figure **E**. Scale bar = 100 μm. **F**. Apoptosis analysis by flow cytometry in AGS and MGC803 with *DLEU1* overexpression or *DLEU1* overexpression combined with ASCC2 knockdown. *, *P* < 0.05; **, *P* < 0.01; ***, *P* < 0.001; ns, not significant

To elucidate the role of *DLEU1* in GC, we investigated its functional dependence on ASCC2. The CCK-8 proliferation assay revealed that knockdown of ASCC2 impaired the pro-proliferative effect of *DLEU1* overexpression in MGC803 and AGS cells (Fig. 5A). Given the established role of ASCC2 in DNA damage response, we further explored whether *DLEU1* exerts its effects on DNA damage repair through ASCC2. Comet assay demonstrated that *DLEU1* overexpression reduced comet tail formation, indicative of enhanced DNA repair, whereas ASCC2 depletion partially restored DNA damage levels (Fig. 5B). Moreover, γ-H2AX was significantly upregulated upon *DLEU1* overexpression, while ASCC2 knockdown mitigated this effect (Fig. 5C). Immunofluorescence staining of γ-H2AX following etoposide treatment further confirmed that ASCC2 depletion delayed the resolution of DNA damage in *DLEU1*-overexpressing cells (Fig. 5D–E). Apoptosis assays further revealed that knockdown of ASCC2 partially restored the apoptotic response in *DLEU1*-overexpressing cells (Fig. 5F). Collectively, these findings suggest that *DLEU1* fulfills its oncogenic function in GC by promoting DNA damage repair in an ASCC2-dependent manner, thereby enhancing cell survival and proliferation.

### *DLEU1* promotes antioxidant function remodeling in GC *via* G6PD regulation

Our findings indicate that *DLEU1* may drive the initiation and progression of GC by modulating G6PD. To further elucidate the role of G6PD in GC, we overexpressed G6PD in AGS and MGC803 cells *via* transfection with a cDNA plasmid or a control vector (Supplementary Fig. 4A-B). G6PD overexpression significantly enhanced cell proliferation, whereas its knockdown inhibited these processes (Supplementary Fig. 4C).

**Fig. 6:**
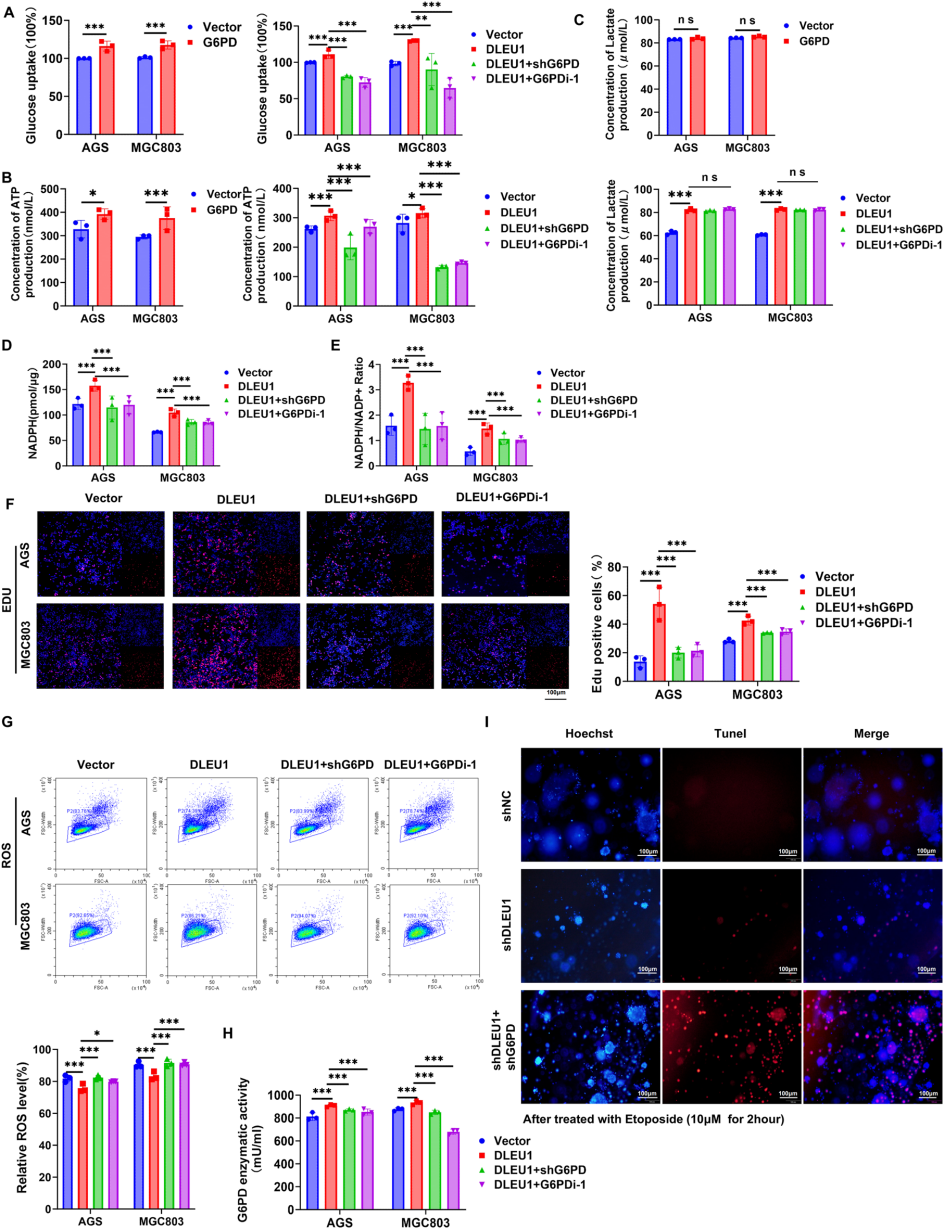
*DLEU1* modulates cellular metabolism, redox homeostasis, and DNA Damage *via* G6PD regulation. **A**-**C**. Glucose uptake, ATP production, lactate production in AGS and MGC803 cells transfected with a G6PD overexpression vector (left) or *DLEU1* overexpression combined with G6PD knockdown (*shG6PD*) or G6PD inhibition (G6PDi-1) (right). **D**-**G**. NADPH levels, NADPH/NADP + ratio, EdU incorporation and ROS levels in AGS and MGC803 cells with *DLEU1* overexpression combined with *shG6PD*, or G6PDi-1 treatment. Scale bar = 100 μm. **H**. G6PD enzymatic activity assay in AGS and MGC803 cells with *DLEU1* overexpression combined with *shG6PD*, or G6PDi-1 treatment. **I**. TUNEL staining of organoids treated with shNC, sh*DLEU1*, and sh*DLEU1* + *shG6PD*. Scale bar = 100 μm. *, *P* < 0.05; **, *P* < 0.01; ***, *P* < 0.001; ns, not significant. See also Figures S4

We observed that G6PD overexpression promoted glucose uptake and ATP production, while it exerted minimal effects on lactate generation (Fig. 6A-C). As the rate-limiting enzyme of the pentose phosphate pathway, G6PD catalyzes the conversion of glucose-6-phosphate into NADPH, thereby maintaining intracellular redox homeostasis. In AGS and MGC803 cells with *DLEU1* overexpression, NADPH levels, the NADPH/NADP⁺ ratio (Fig. 6D, E), and DNA synthesis (Fig. 6F) were significantly increased, while ROS levels (Fig. 6G) were markedly reduced. However, G6PD knockdown or inhibition with G6PD inhibitor (G6PDi-1) in *DLEU1*-overexpressing GC cells effectively reversed these effects (Fig. 6D-G). Moreover, *DLEU1* overexpression significantly enhanced G6PD enzymatic activity, an effect that was suppressed upon G6PD knockdown or G6PDi-1 treatment (Fig. 6H). Additionally, knockdown of G6PD or G6PDi-1 treatment exacerbated comet tail formation, suggesting that increased ROS levels may compromise DNA damage repair (Supplementary Fig. 4D). To further investigate the functional link between *DLEU1* silencing and chemo-sensitivity, we assessed apoptosis markers in our organoid model. TUNEL staining revealed that *DLEU1* knockdown significantly enhanced Etoposide-induced apoptosis compared to the *shNC* control. This pro-apoptotic effect was further amplified by concurrent G6PD knockdown, indicating that *DLEU1* silencing potentiates chemotherapy-induced cell death through mitochondrial apoptotic pathway activation (Fig. 6I). Collectively, these results demonstrate that *DLEU1* promotes antioxidant remodeling in GC cells, at least in part, through the regulation of G6PD.

### Targeting the *DLEU1*/ASCC2/G6PD axis restrains GC proliferation in *vivo*

**Fig. 7:**
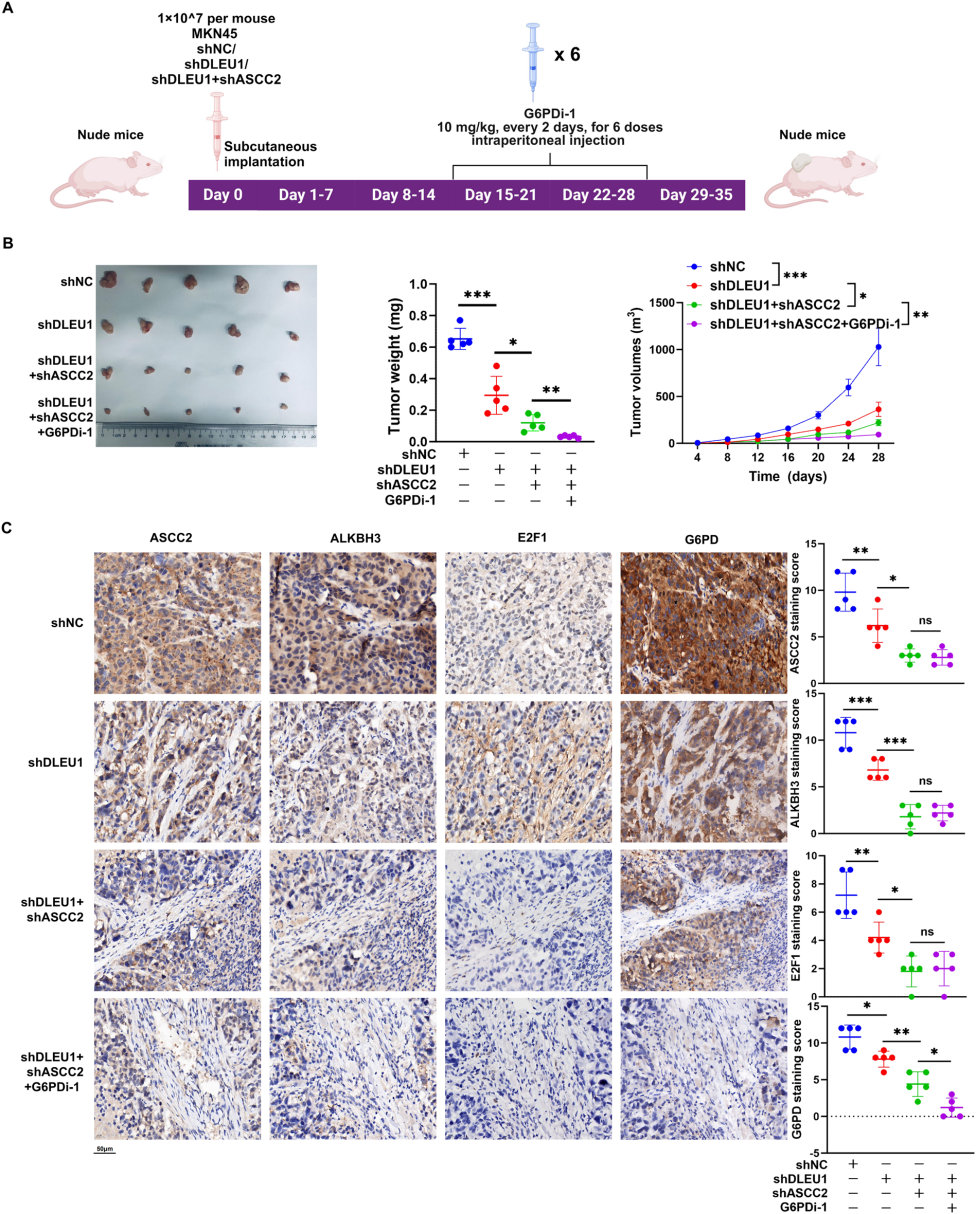
*DLEU1* knockdown suppresses tumor growth by targeting ASCC2 and G6PD in xenograft models. **A**. Schematic representation of the xenograft experiment. MKN45 cells with stable knockdown of *DLEU1* (*shDLEU1*) or co-knockdown of *DLEU1* and ASCC2 (*shDLEU1* + *shASCC2*) were subcutaneously implanted into nude mice. G6PD inhibitor (G6PDi-1) was administered intraperitoneally (10 mg/kg, every 2 days, for a total of six doses) from day 15 to day 21. **B**. Tumor growth analysis across different groups. Left: Representative images of tumors from the indicated groups. Middle: Tumor weight comparison among groups. Right: Tumor volume progression over time. **C**. IHC staining for ASCC2, ALKBH3, E2F1, and G6PD in tumor tissues. Representative IHC images (left) and quantification of staining scores (right) for each marker. Scale bar = 50 μm. *, *P* < 0.05; **, *P* < 0.01; ***, *P* < 0.001; ns, not significant

To further explore the therapeutic potential of targeting the *DLEU1*/ASCC2/G6PD axis in GC, we established stable MKN45 cell lines transduced with *shNC*, *shDLEU1*, or *shDLEU1* in combined with *shASCC2* and subcutaneously implanted them into nude mice. Fourteen days after tumor formation, a subset of mice in the *shDLEU1* + *shASCC2* group received additional treatment with G6PDi-1 (10 mg/kg) administered every other day for a total of six doses (Fig. 7A). Tumor analysis revealed a significant reduction in tumor growth across all experimental groups compared to the *shNC* group, with more pronounced inhibitory effects observed upon combined suppression of multiple targets (Fig. 7B). Furthermore, the progressive suppression of ASCC2, ALKBH3, E2F1, and G6PD led to a marked decrease in their immunohistochemical staining scores, indicating reduced protein expression and altered cellular localization within the tumor microenvironment (Fig. 7C). These findings correlated with the observed decrease in tumor growth and proliferation, further highlighting the critical role of targeting multiple key components of the *DLEU1*/ASCC2/G6PD axis in modulating tumor biology.

### Clinical significance of the *DLEU1*/ASCC2/ALKBH3/E2F1 axis and its epigenetic regulation in GC

**Fig. 8:**
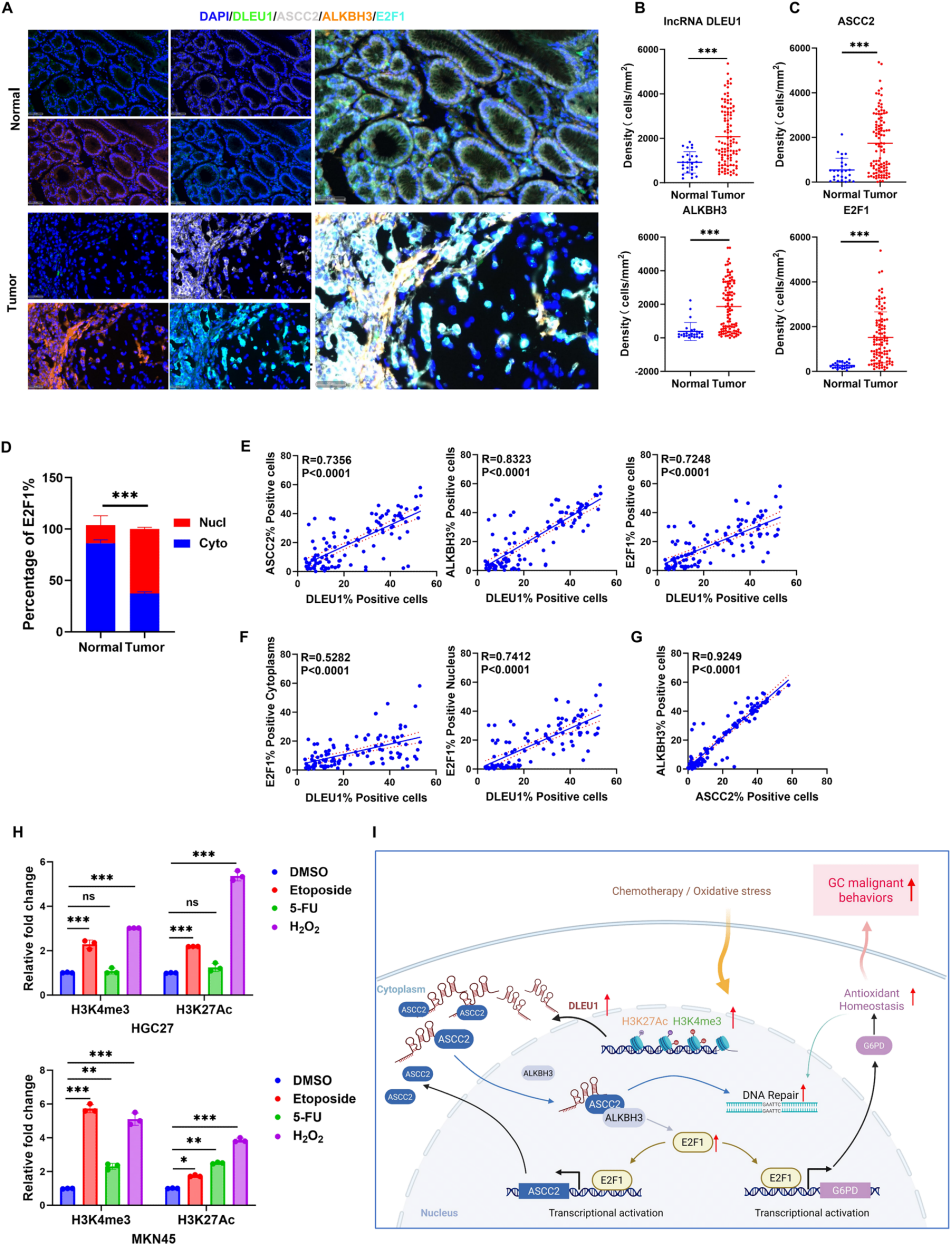
Expression and correlation analysis of *DLEU1* and associated markers in normal and GC tissues, and its epigenetic regulation in GC. **A**. Representative multiplex immunofluorescence staining images showing *DLEU1* (green), ASCC2 (white), ALKBH3 (orange), and E2F1 (cyan) in normal and tumor tissues (*N* = 26, T = 104). Scale bar = 50 μm. **B**. Quantification of *DLEU1*-positive cell density in normal and tumor tissues. **C**. Quantification of ASCC2-, ALKBH3-, and E2F1-positive cell densities in normal and tumor tissues. **D**. Percentage of E2F1 localization in the nucleus and cytoplasm in normal and tumor tissues. **E**. Scatter plots showing positive correlations between *DLEU1* expression and ASCC2, ALKBH3, and E2F1 expression in tumor tissues. **F**. Correlation between *DLEU1* expression and E2F1 localization in the cytoplasm and nucleus in tumor tissues. **G**. Correlation between ASCC2- and ALKBH3-positive cell in tumor tissues. **H**. HGC27 and MKN45 cells were treated with DMSO (control), Etoposide (10 µM, 24 h), 5-Fluorouracil (5-FU, 20 µg/mL, 24 h), or H_2_O_2_ (10 µM, 2 h). The enrichment of H3K4me3 and H3K27ac at the *DLEU1* promoter was analyzed by ChIP-qPCR. **I**. Proposed model illustrating how *DLEU1* promotes gastric cancer progression *via* the ASCC2/ALKBH3/E2F1/G6PD axis, impacting transcriptional activation, DNA repair, and redox homeostasis. *, *P* < 0.05; **, *P* < 0.01; ***, *P* < 0.001; ns, not significant

To investigate the clinical significance of the *DLEU1*/ASCC2/ALKBH3/E2F1 signaling pathway in GC, we simultaneously examined the *DLEU1* mRNA expression and the protein expression levels of ASCC2, ALKBH3 and E2F1 in tumor and normal gastric tissues using mIHC staining on tissue microarrays (TMA; *N* = 26, T = 104; Fig. 8A). Quantitative analysis revealed a significantly higher proportion of *DLEU1*-positive cells in GC tissues compared to matched normal gastric tissues (*P* < 0.001, Fig. 8B). Similarly, the densities of ASCC2-positive, ALKBH3-positive, and E2F1-positive cells were also significantly higher in GC tissues (Fig. 8C, all *P* < 0.001), with evident co-localization among these markers. Notably, analysis of E2F1 subcellular localization demonstrated a significantly higher nuclear-to-cytoplasmic ratio in tumor tissues relative to normal tissues (Fig. 8D, *P* < 0.001), suggesting transcriptional activation of E2F1 in GC. Correlation analysis demonstrated strong positive associations between *DLEU1* and ASCC2 (*R* = 0.7356, *P* < 0.0001), ALKBH3 (*R* = 0.8323, *P* < 0.0001), and E2F1 (*R* = 0.7248, *P* < 0.0001; Fig. 8E). Moreover, a stronger positive correlation was observed between *DLEU1* and nuclear-localized E2F1 (*R* = 0. 7412, *P* < 0.0001), whereas the correlation with cytoplasmic E2F1 was comparatively weaker (*R* = 0.5282, *P* < 0.0001; Fig. 8F). Additionally, the robust correlation between ASCC2 and ALKBH3 further supported their close functional interplay (*R* = 0.9249, *P* < 0.0001). These findings highlight the clinical relevance of the *DLEU1*/ASCC2/ALKBH3/E2F1 signaling pathway in GC (Fig. 8I).

To estimate whether these histone marks are dynamically regulated in response to environmental stimuli, such as chemotherapy or oxidative stress. We treated gastric cancer cell lines HGC27 and MKN45 with the chemotherapeutic agents Etoposide (10 µM, 24 h) and 5-Fluorouracil (5-Fu, 20 µg/mL, 24 h), as well as the oxidative stress inducer H_2_O_2_ (10 µM, 2 h). Using ChIP-qPCR analysis, we assessed the enrichment of H3K4me3 and H3K27Ac at the *DLEU1* promoter under these conditions, with a DMSO-treated group serving as the control (Fig. 8H). This suggests that the hostile microenvironment induced by chemotherapy or oxidative stress may dynamically enhance the active histone marks at the *DLEU1* locus. This could potentially establish a positive feedback loop that further amplifies *DLEU1* expression, possibly contributing to tumor cell adaptation or resistance.

## Discussion

LncRNAs play critical roles in GC progression [32], particularly in regulating DNA repair and metabolic reprogramming. Previous studies have demonstrated that lncRNAs orchestrate these processes by modulating key molecular pathways; for instance, *SNHG17*-mediated *miR-3909*/RING1/Rad51 axis altered the DNA repair system [33], while CCAT1-PTBP1 interactions facilitated glycolysis in GC [34]. Beyond these roles, lncRNAs are also critical regulators of oxidative stress, enabling cancer cells to maintain redox homeostasis and resist oxidative damage. Notably, *LncRNA-HMG* promotes chemoresistance in colorectal cancer by inhibiting p53-mediated ferroptosis and enhancing ROS scavenging capacity [35].

In the present study, we identified *DLEU1* as a multifunctional regulator that simultaneously modulates DNA repair, oxidative stress response, and glucose metabolism in GC. Clinically, we demonstrated that *DLEU1* is epigenetically activated by H3K27 acetylation and H3K4 methylation, driving its overexpression in GC tissues. Mechanistically, we uncovered a previously unrecognized axis in which *DLEU1* facilitates ASCC2 nuclear translocation and interaction with ALKBH3, thereby stabilizing E2F1 mRNA and subsequently amplifying G6PD transcription (Fig. 8I). This axis orchestrates NADPH production, redox homeostasis, and glucose metabolism, while synergistically enhancing DNA repair. Functionally, dual silencing of *DLEU1* and ASCC2, in combination with G6PD inhibition, synergistically suppressed the viability of GC cells and tumor growth in vivo, highlighting the therapeutic potential of targeting the *DLEU1*/ASCC2/G6PD axis. Notably, our findings revealed that chemotherapy and oxidative stress dynamically enhance active histone marks at the *DLEU1* promoter, thereby establishing a positive feedback loop that amplifies *DLEU1* expression and potentially contributes to tumor adaptation. This mechanism is reminiscent of ultrasensitive gene regulation through histone modification feedback loops.

While prior work established *DLEU1* as an oncogenic lncRNA promoting GC proliferation *via* epigenetic mechanisms [11, 12], our study unveils novel regulatory layers. We illustrate a dual regulatory mechanism in which lncRNAs simultaneously influence the nuclear translocation of DNA repair complexes (ASCC2) and the transcriptional activation of metabolic enzymes (G6PD). Notably, building upon the seminal work by Wang et al. demonstrating ALKBH3-mediated stabilization of E2F1 mRNA through m1A demethylase activity [30], we confirmed that ALKBH3 overexpression robustly elevates E2F1 protein levels, aligning with established mechanisms. Given the prior rigorous validation of m1A modification sites and RNA decay dynamics in E2F1 regulation, our study pivots to delineate downstream metabolic networks. In addition, our cell-based assays revealed that E2F1 directly binds to the G6PD promoter and drives its transcriptional activation, thereby rewiring the PPP flux. This finding extends the functional scope of the ALKBH3–E2F1 axis to the regulation of redox homeostasis. Furthermore, as a classical cell cycle regulator [36], E2F1 preferentially regulates the PPP [37, 38]. This division of labor may reflect the distinct metabolic demands at different cell cycle phases, E2F1-driven G1/S transition requires a high supply of nucleotide precursors, and the PPP serves as a key metabolic hub by providing both ribose-5-phosphate and NADPH to support this process [39]. Additionally, we established a positive feedback loop in which E2F1 transcriptionally regulates ASCC2, further amplifying the oncogenic signaling mediated by *DLEU1*. This finding provides new insights into the intricate regulatory network governing DNA repair and metabolic adaptation in GC.

Despite the novel insights provided by this study, several limitations should be acknowledged. First, as this study is based on a retrospective cohort analysis, given the racial heterogeneity and epidemiological differences, the observed correlation between *DLEU1* expression and clinical outcomes in Chinese GC population requires further validation in more multicenter cohorts. Second, while we have demonstrated E2F1-mediated transcriptional regulation of G6PD, the precise stoichiometric relationship and temporal dynamics of this regulation in vivo remain unclear. Given the ubiquitous and high basal expression of G6PD in both tumor and normal gastric tissues, establishing a clear regulatory link between E2F1 and G6PD in patient-derived samples is technically challenging. Bulk tissue-level analyses may be confounded by stromal or epithelial background signals. Therefore, we relied on cell-based assays to validate E2F1-driven transcriptional activation of G6PD. Future studies employing spatial transcriptomics or single-cell approaches may help overcome these limitations. Third, while our data establish *DLEU1* as a key regulator, the question of functional redundancy with other lncRNAs (e.g., *PVT1* or *SNHG17*) arises. Transcriptomic analyses suggest that these lncRNAs exhibit distinct expression patterns, and silencing *DLEU1* alone significantly impairs DNA repair, supporting a non-redundant role. Future studies combining multiple lncRNA perturbations could elucidate potential synergistic effects.

Clinically, we observed elevated *DLEU1* expression in MSI-H and intestinal-type GC, suggesting its potential as a molecular classifier. Notably, although the MSI-H phenotype typically arises from functional deficiencies in the MMR system, our exploratory analyses revealed that *DLEU1* overexpression upregulates key MMR genes, such as MLH1, MSH6, and PMS2. This seemingly paradoxical observation may imply the existence of a compensatory feedback mechanism within MSI-H gastric cancer or reflect intratumoral heterogeneity. The precise role of *DLEU1* in MMR proficiency warrants further investigation using CRISPR/Cas9-based gene editing in MMR-deficient models to elucidate the underlying mechanism.

Despite uncovering a critical role for *DLEU1* in GC progression, several key scientific questions remain to be addressed to further elucidate its mechanism and facilitate clinical translation. First, although chromatin isolation by RNA purification coupled with mass spectrometry (ChIRP-MS) identified ASCC2 as a *DLEU1*-interacting protein, the specific binding domains and dynamic regulatory mechanisms remain unclear. While the scaffolding function of *DLEU1* in facilitating the ASCC2-ALKBH3 interaction is established by RNA pull-down and RIP assays, the specific structural domains responsible for this activity remain uncharacterized. A comprehensive mapping of these functional elements, such as through RNA truncation analysis, is considered technically challenging due to the complex secondary and tertiary structures anticipated for this long lncRNA. The precise delineation of *DLEU1*’s functional domains is identified as a critical objective for future research. Second, whether the *DLEU1*-ASCC2-G6PD axis is subject to external regulatory signals remains to be explored, yet it is still unclear whether this regulation is dynamically influenced by DNA damage signals (e.g., ATM/ATR kinases [40]) or metabolic stress (e.g., the NADPH/NADP + ratio). In this context, the observed DNA damage upon G6PD inhibition in *DLEU1*-overexpressing cells may involve multiple mechanisms. G6PD is a critical enzyme in the pentose phosphate pathway, serving as a primary source of NADPH, which is essential for maintaining redox homeostasis and providing ribose-5-phosphate for nucleotide synthesis. The increase in DNA damage markers may be attributed to elevated oxidative stress due to insufficient NADPH or imbalances in dNTP pools. While our data cannot distinguish the relative contributions, both pathways likely act synergistically, and future studies should directly quantify ROS and dNTP dynamics.

## Conclusions

Taken together, this study establishes *DLEU1* as a multifunctional lncRNA that integrates epigenetic, genomic, and metabolic reprogramming in GC *via* the ASCC2/ALKBH3-E2F1-G6PD axis. These findings provide a conceptual framework for understanding how non-coding RNAs orchestrate multidimensional cancer hallmarks, a paradigm that may extend to other malignancies. Targeting the *DLEU1*/ASCC2/G6PD axis significantly suppresses GC cells proliferation and tumor growth, proposing a therapeutic strategy targeting this pathway. Although therapeutic targeting of lncRNAs remains challenging, our study identifies actionable targets for combination therapy and underscores the importance of dissecting lncRNA functions within complex regulatory networks to develop effective treatment strategies.

## Supplementary Information

Below is the link to the electronic supplementary material.

Supplementary Material 1

Supplementary Material 2

Supplementary Material 3

## Data Availability

Data will be made available on request.

## Abbreviations

GC: Gastric cancer
lncRNA: Long non-coding RNA
NEAT1: Nuclear-enriched abundant transcript 1
DDX5: DEAD-box helicase 5
PVT1: Plasmacytoma variant translocation 1
FOXM1: Forkhead box M1
DLEU1: Deleted in Lymphatic Leukemia 1
LSD1: Lysine-specific demethylase 1
SMYD2: SET and MYND domain containing 2
ASCC2: Activating Signal Cointegrator 1 Complex Subunit 2
ALKBH3: AlkB homolog 3
E2F1: E2F Transcription Factor 1
G6PD: Glucose-6-phosphate dehydrogenase
FUSCC: Fudan University Shanghai Cancer Center
TMA: Tissue microarrays
mIHC: multiplex Immunohistochemistry
TSA: Tyramide signal amplification
FFPE: Formalin-fixed paraffin-embedded
HIER: Heat-induced epitope retrieval
BSA: Bovine serum albumin
PI: Propidium iodide
ChIRP: Chromatin Isolation by RNA Purification
OS: Overall survival
DFS: Disease-free survival
TCGA: The Cancer Genome Atlas
MSI-H: High microsatellite instability
LNM: Lymphatic metastasis
ROC: Receiver operating characteristic
ECAR: Extracellular acidification rate
OCR: Oxygen consumption rate
LC/MS: Liquid Chromatography-Mass Spectrometry
CHX: Cycloheximide
PPP: Pentose phosphate pathway
MMR: Mismatch repair
