# Supplementary material for: Histone modification-regulated LncRNA DLEU1 interacts with ASCC2/ALKBH3 complex to drive DNA repair, antioxidant homeostasis and glucose metabolism in gastric cancer
Source: Biomark Res. 2026 Jan 2;14:1. doi: 10.1186/s40364-025-00867-y (PMC12764130; doi:10.1186/s40364-025-00867-y)
Supplement: Supplementary file 2 — Supplementary Material 2 [file 40364_2025_867_MOESM2_ESM.pdf]

Fig 1I

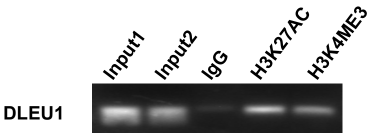
Input1
Input2
IgG
H3K27AC
H3K4ME3
DLEU1

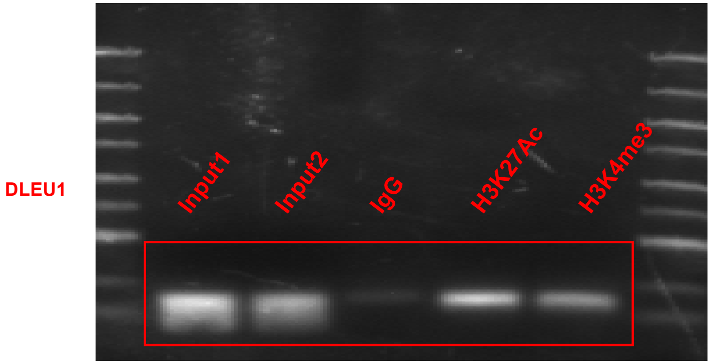
DLEU1
Input1
Input2
IgG
H3K27Ac
H3K4me3

Fig 2E

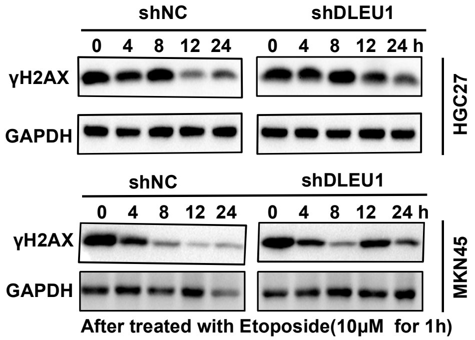
shNC
shDLEU1
0 4 8 12 24
0 4 8 12 24 h
γH2AX
GAPDH
HGC27
shNC
shDLEU1
0 4 8 12 24
0 4 8 12 24 h
γH2AX
GAPDH
MKN45
After treated with Etoposide(10μM for 1h)

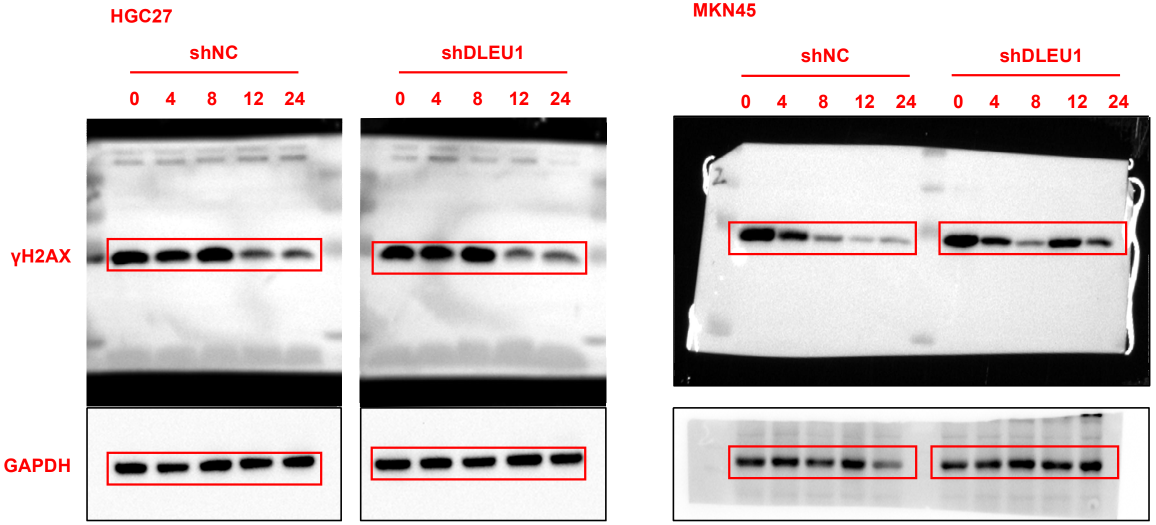
HGC27
shNC
shDLEU1
0 4 8 12 24
0 4 8 12 24
γH2AX
GAPDH
MKN45
shNC
shDLEU1
0 4 8 12 24
0 4 8 12 24

Fig1G

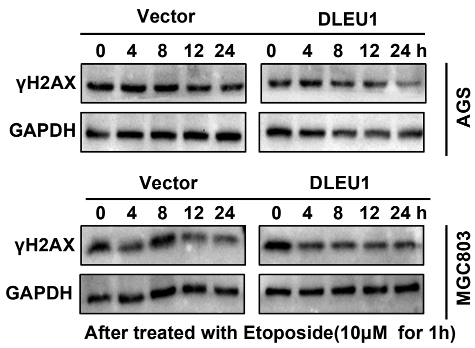
Vector
DLEU1
0 4 8 12 24
0 4 8 12 24 h
γH2AX
GAPDH
AGS
Vector
DLEU1
0 4 8 12 24
0 4 8 12 24 h
γH2AX
GAPDH
MGC803
After treated with Etoposide(10μM for 1h)

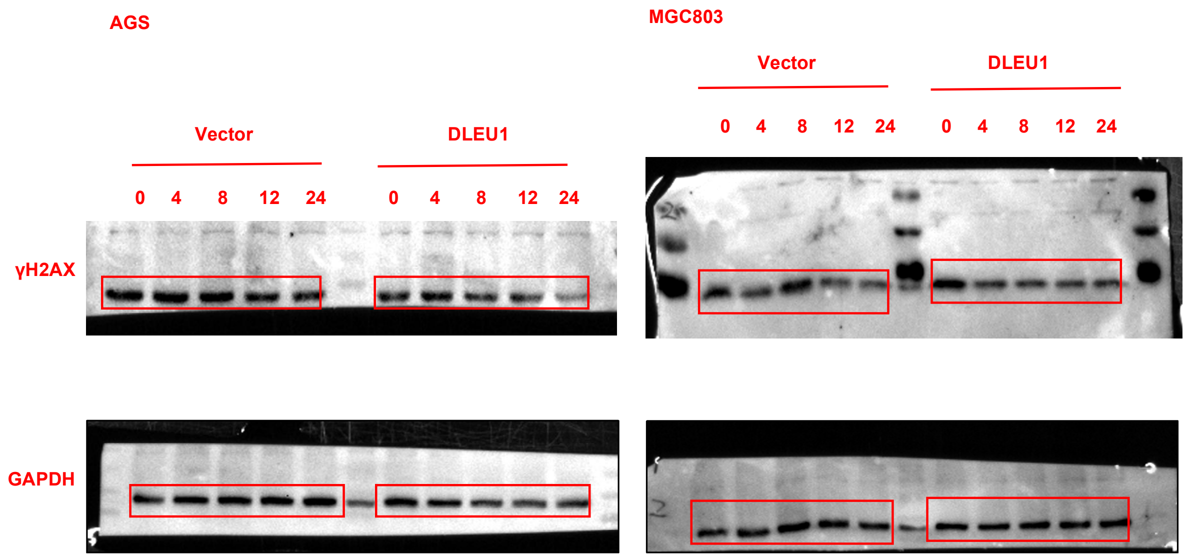
AGS
MGC803
Vector
DLEU1
0 4 8 12 24
0 4 8 12 24
Vector
DLEU1
0 4 8 12 24
0 4 8 12 24
γH2AX
GAPDH

Fig3B

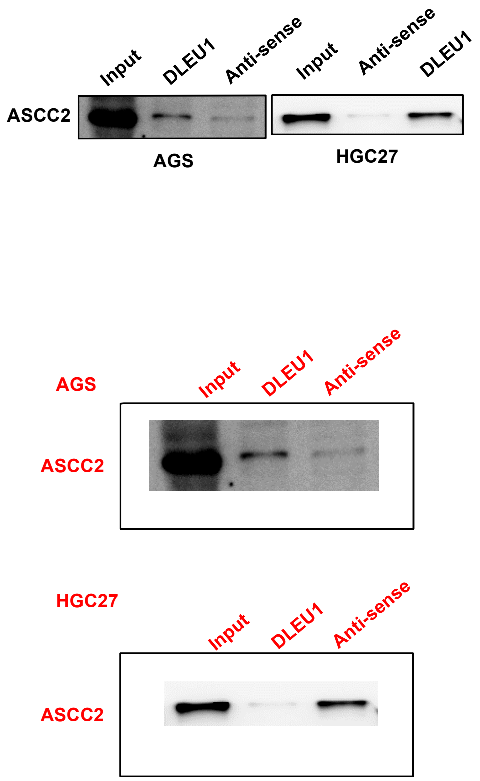

Input
DLEU1
Anti-sense
Input
Anti-sense
DLEU1
ASCC2
AGS
HGC27
AGS
Input
DLEU1
Anti-sense
ASCC2
HGC27
Input
DLEU1
Anti-sense
ASCC2

Fig3E

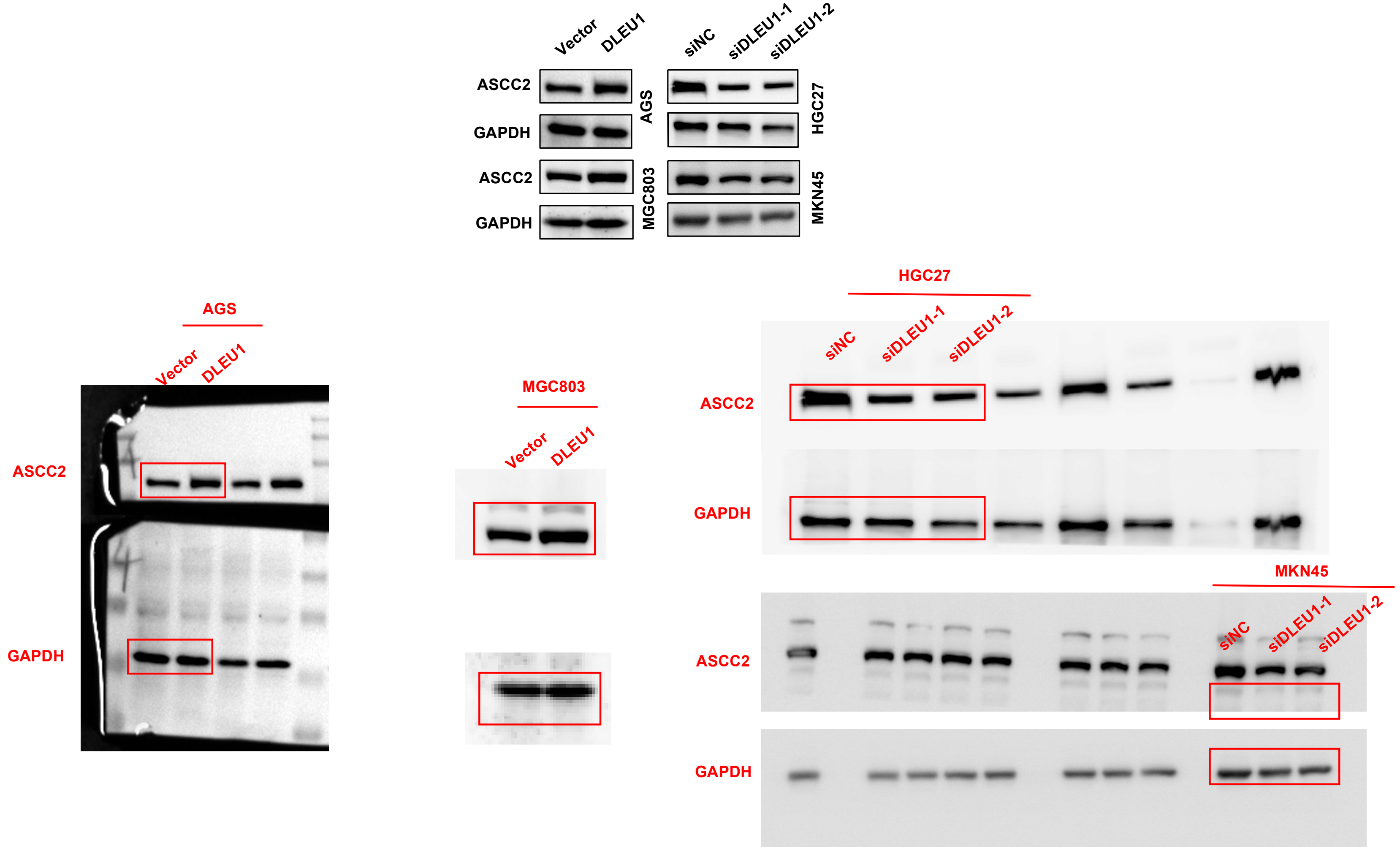

Vector
DLEU1
siNC
siDLEU1-1
siDLEU1-2
ASCC2
GAPDH
ASCC2
GAPDH
AGS
HGC27
MGC803
MKN45
AGS
Vector
DLEU1
ASCC2
GAPDH
MGC803
Vector
DLEU1
HGC27
siNC
siDLEU1-1
siDLEU1-2
ASCC2
GAPDH
MKN45
siNC
siDLEU1-1
siDLEU1-2
ASCC2
GAPDH

Fig3F

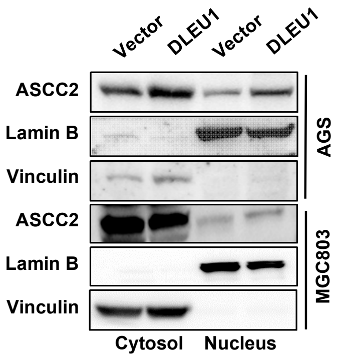

Vector
DLEU1
Vector
DLEU1
ASCC2
Lamin B
Vinculin
ASCC2
Lamin B
Vinculin
AGS
MGC803
Cytosol
Nucleus

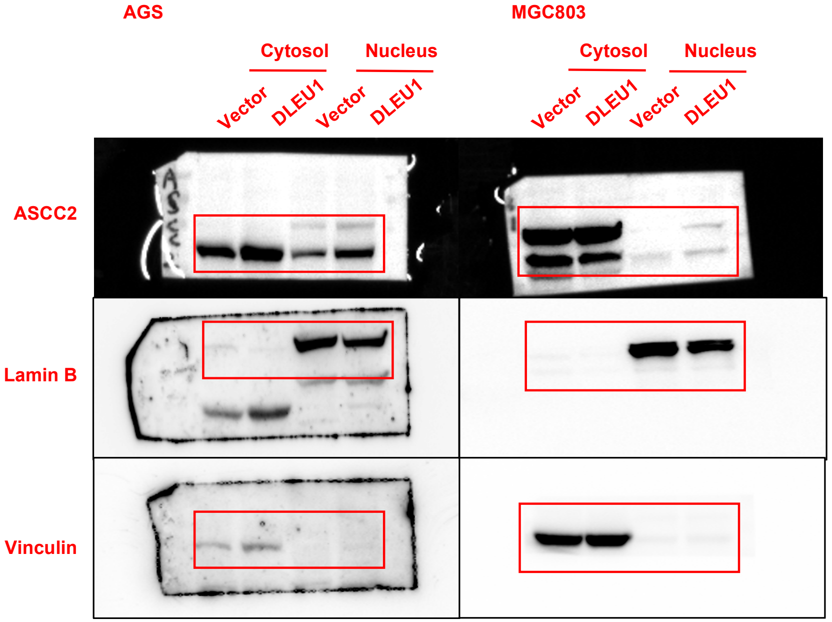

AGS
MGC803
Cytosol
Nucleus
Cytosol
Nucleus
Vector
DLEU1
Vector
DLEU1
Vector
DLEU1
Vector
DLEU1
ASCC2
Lamin B
Vinculin

Fig3H

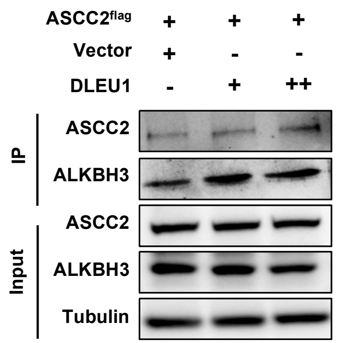

ASCC2flag + + +
Vector + - -
DLEU1 - + ++
IP
ASCC2
ALKBH3
Input
ASCC2
ALKBH3
Tubulin

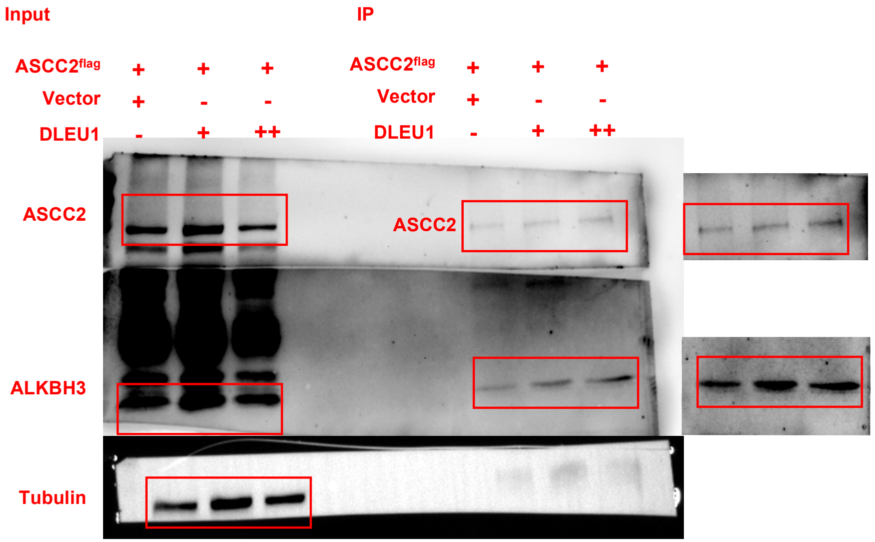

Input
ASCC2flag + + +
Vector + - -
DLEU1 - + ++
ASCC2
ALKBH3
Tubulin
IP
ASCC2flag + + +
Vector + - -
DLEU1 - + ++
ASCC2

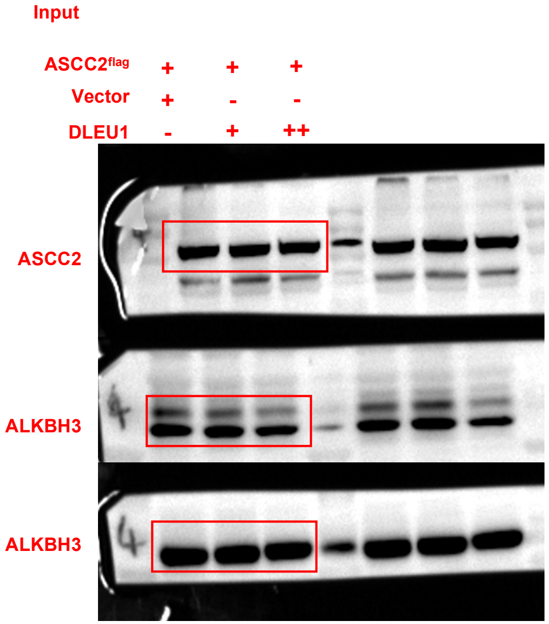

Input
ASCC2flag + + +
Vector + - -
DLEU1 - + ++
ASCC2
ALKBH3
ALKBH3

Fig3I

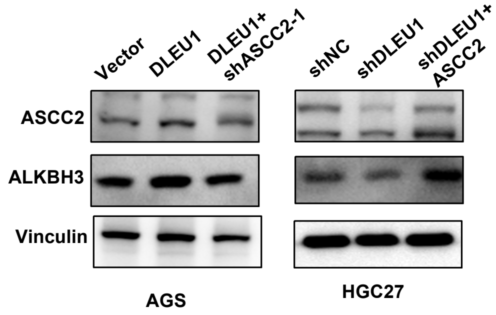
Vector
DLEU1
DLEU1+ shASCC2-1
shNC
shDLEU1
shDLEU1+ ASCC2
ASCC2
ALKBH3
Vinculin
AGS
HGC27

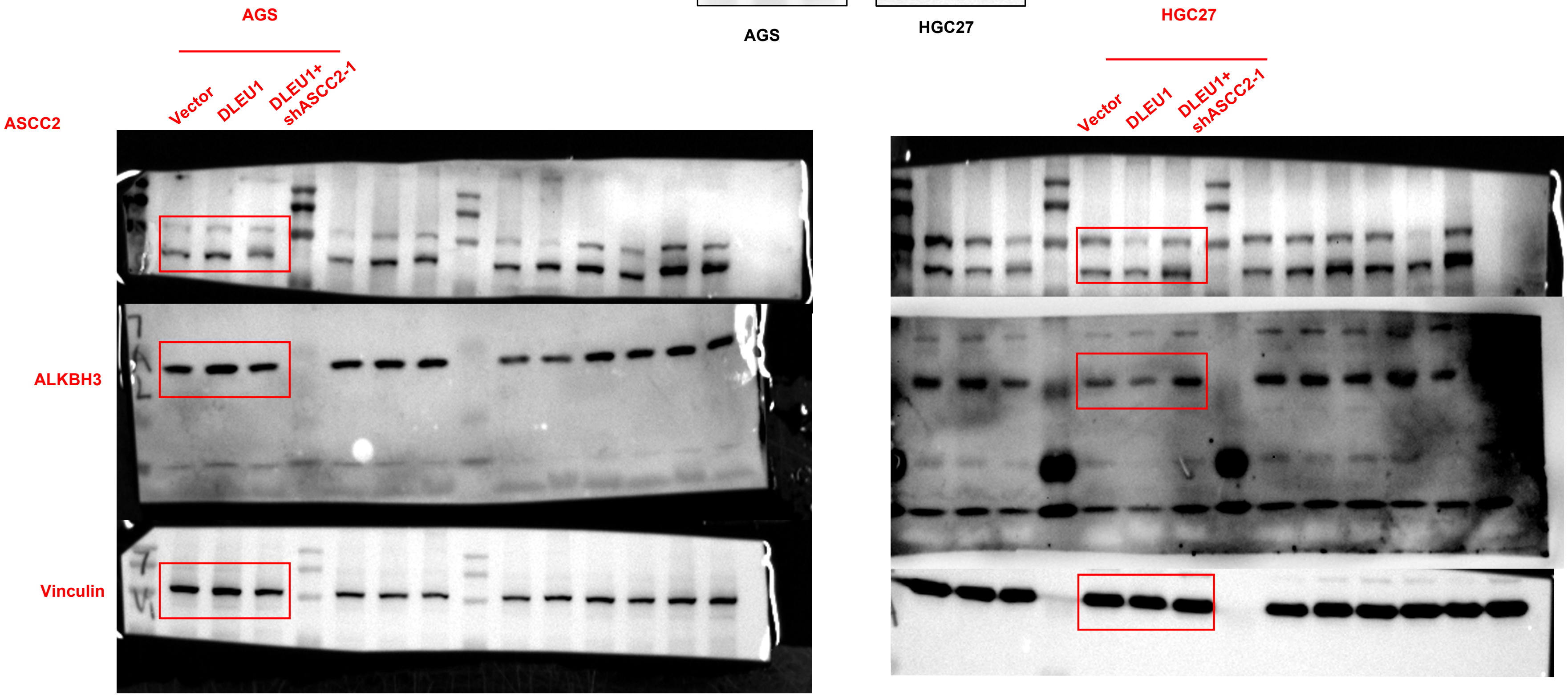
AGS
Vector
DLEU1
DLEU1+ shASCC2-1
ASCC2
ALKBH3
Vinculin
HGC27
Vector
DLEU1
DLEU1+ shASCC2-1

SFig3B

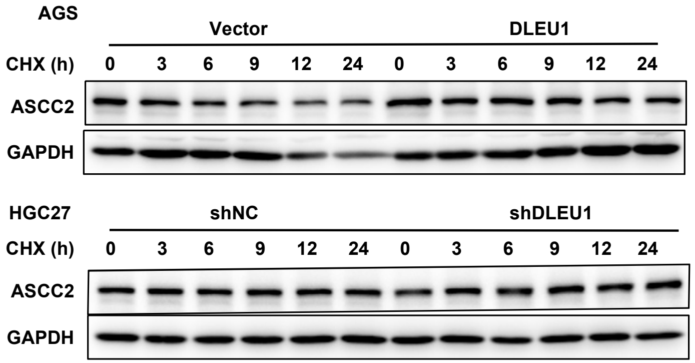
AGS
Vector
DLEU1
CHX (h)
0 3 6 9 12 24 0 3 6 9 12 24
ASCC2
GAPDH
HGC27
shNC
shDLEU1
CHX (h)
0 3 6 9 12 24 0 3 6 9 12 24
ASCC2
GAPDH

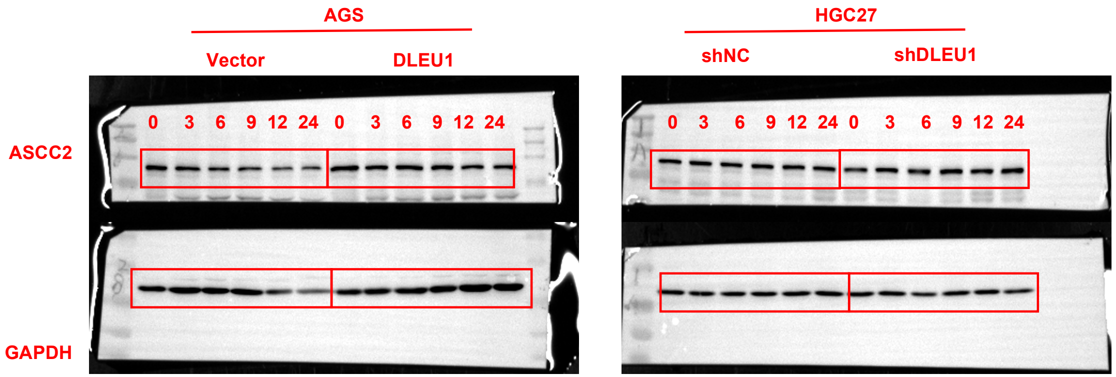
AGS
Vector
DLEU1
ASCC2
0 3 6 9 12 24 0 3 6 9 12 24
GAPDH
HGC27
shNC
shDLEU1
0 3 6 9 12 24 0 3 6 9 12 24

Fig4B

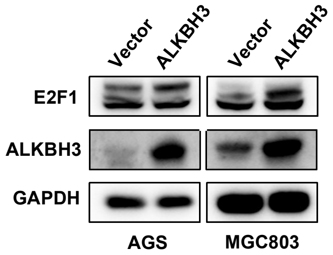
Vector
ALKBH3
Vector
ALKBH3
E2F1
ALKBH3
GAPDH
AGS
MGC803

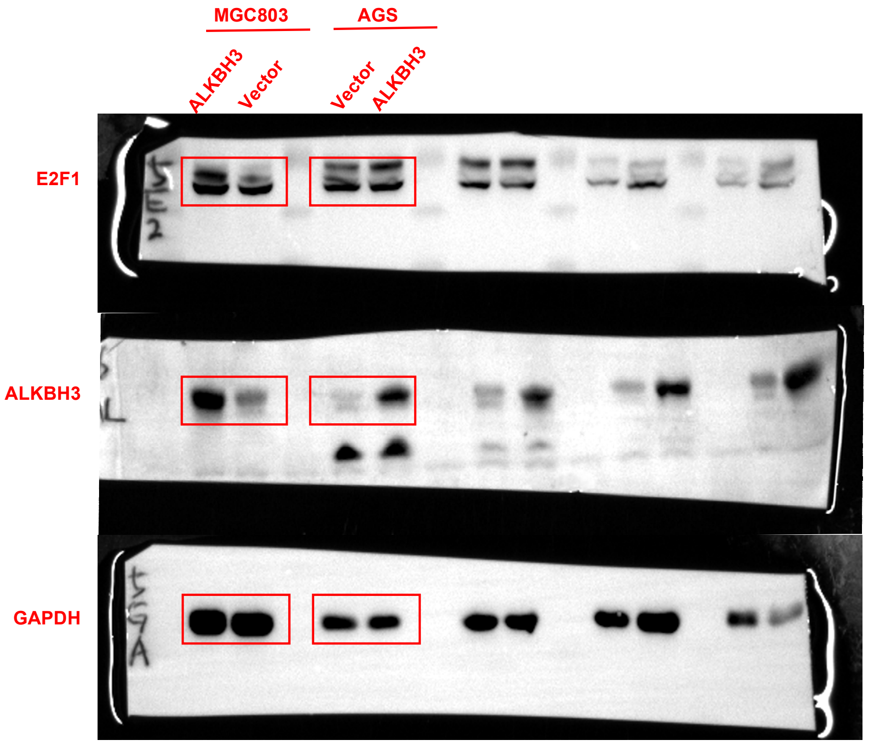
MGC803
AGS
ALKBH3
Vector
Vector
ALKBH3
E2F1
ALKBH3
GAPDH

Fig4E

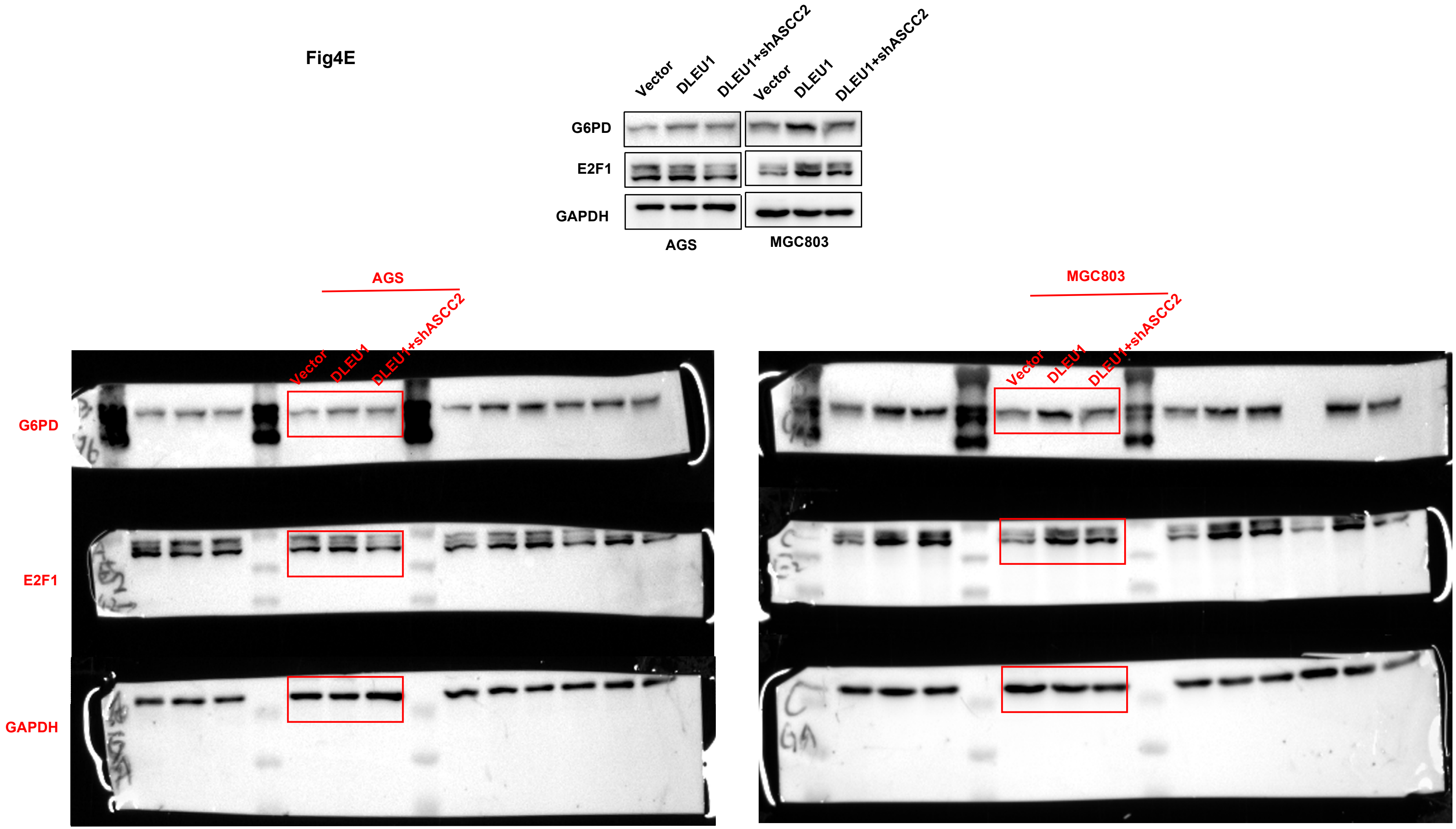
Vector
DLEU1
DLEU1+shASCC2
Vector
DLEU1
DLEU1+shASCC2
G6PD
E2F1
GAPDH
AGS
MGC803
AGS
Vector
DLEU1
DLEU1+shASCC2
MGC803
Vector
DLEU1
DLEU1+shASCC2
G6PD
E2F1
GAPDH

Fig4G

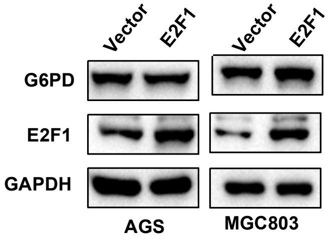

Vector
E2F1
Vector
E2F1
G6PD
E2F1
GAPDH
AGS
MGC803

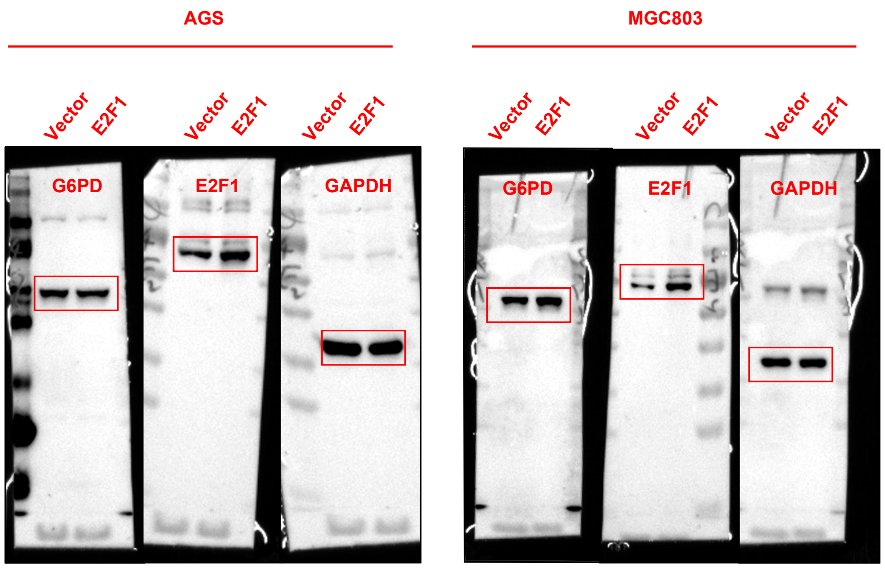

AGS
MGC803
Vector
E2F1
G6PD
E2F1
GAPDH

Fig4I

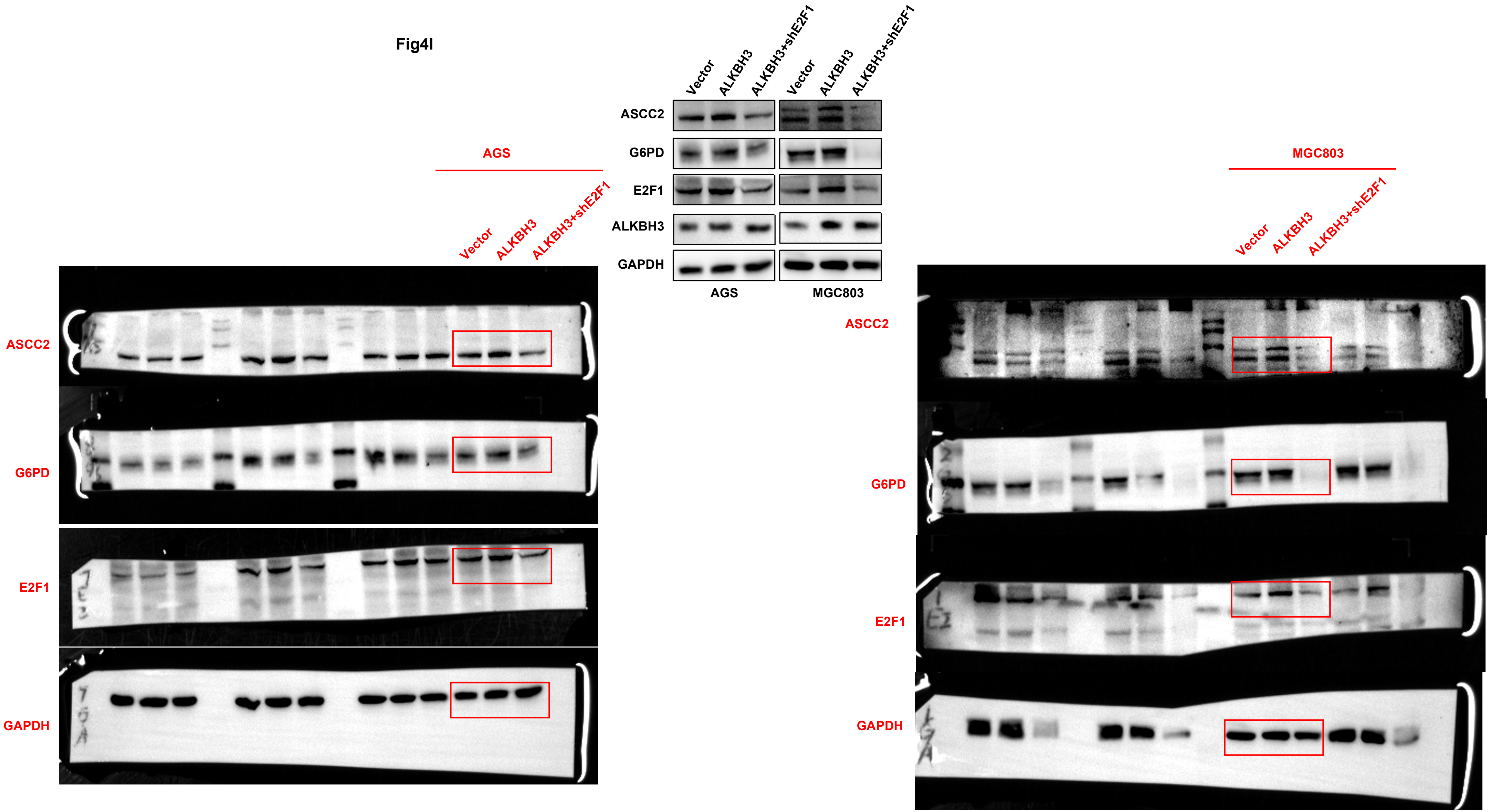

Vector
ALKBH3
ALKBH3+shE2F1
Vector
ALKBH3
ALKBH3+shE2F1
ASCC2
G6PD
E2F1
ALKBH3
GAPDH
AGS
MGC803
AGS
Vector
ALKBH3
ALKBH3+shE2F1
ASCC2
G6PD
E2F1
GAPDH
MGC803
Vector
ALKBH3
ALKBH3+shE2F1
ASCC2
G6PD
E2F1
GAPDH

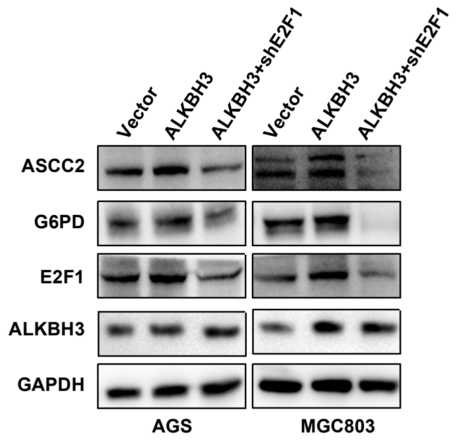
Vector
ALKBH3
ALKBH3+shE2F1
Vector
ALKBH3
ALKBH3+shE2F1
ASCC2
G6PD
E2F1
ALKBH3
GAPDH
AGS
MGC803

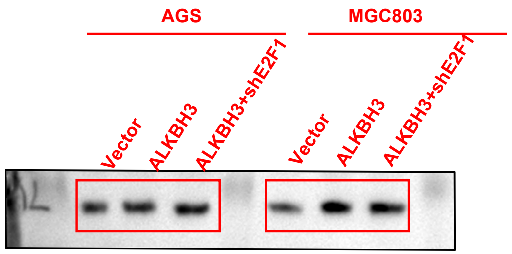
AGS
MGC803
Vector
ALKBH3
ALKBH3+shE2F1
Vector
ALKBH3
ALKBH3+shE2F1

Fig4I

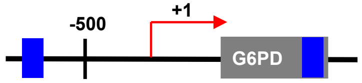

Consensus for E2F1 binding: TTTNNCG
Promoter Site: GCGTAAAA (-873/-866); TTTAACGA(-606/-598);
CCGAGAAA(-225/-218); TTTCGCGG(-209/-202)
Inton2 Site: GAAAAA(187-192); TTTCGCGG(199/205)

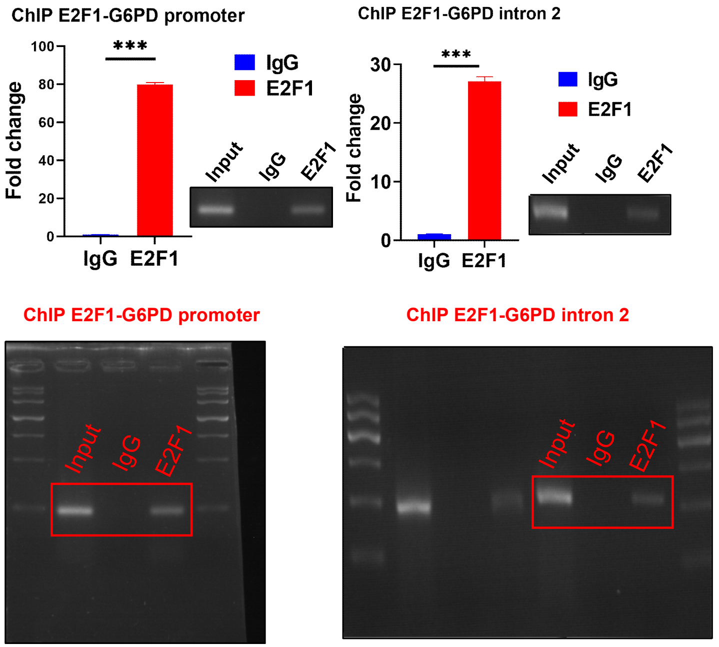

Fig4L

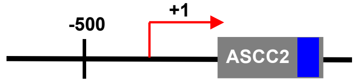

Consensus for E2F1 binding: GCGSSA
Intron Sites: CTTGTCGCCTT(87-97),
CTTCCTGCGTT(110-120),
TCTCGCGGCTC(130-140),
GCTGCCGGCCA(158-168)

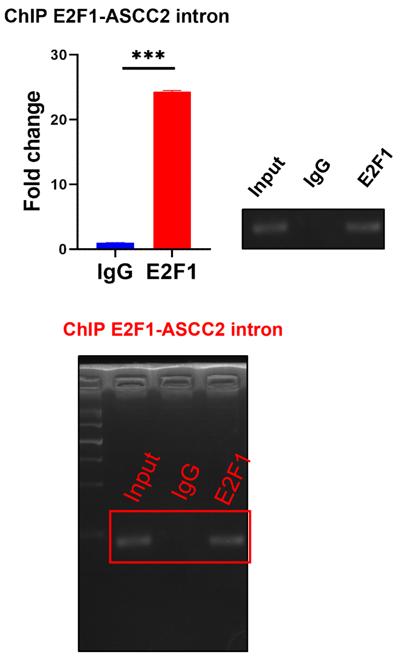

Fig5C

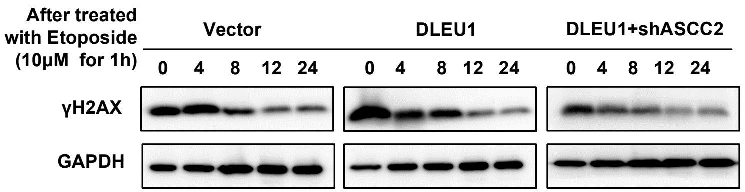
After treated
with Etoposide
(10μM for 1h)
Vector
DLEU1
DLEU1+shASCC2
0 4 8 12 24
0 4 8 12 24
0 4 8 12 24
γH2AX
GAPDH

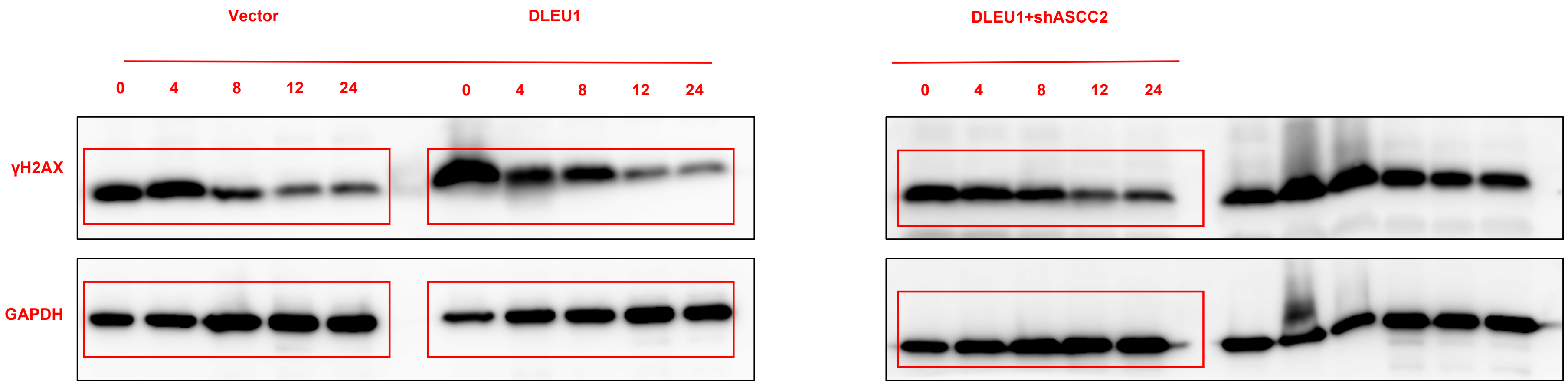
Vector
DLEU1
DLEU1+shASCC2
0 4 8 12 24
0 4 8 12 24
0 4 8 12 24
γH2AX
GAPDH

SFig4A

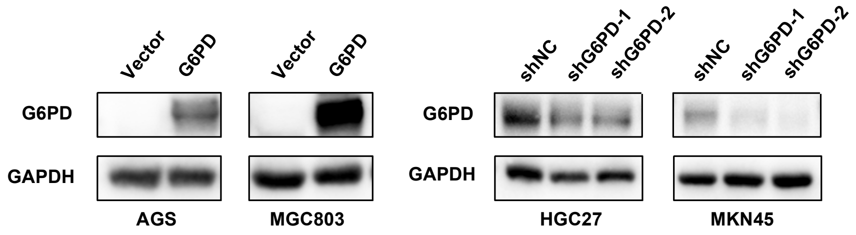
Vector
G6PD
Vector
G6PD
shNC
shG6PD-1
shG6PD-2
shNC
shG6PD-1
shG6PD-2
G6PD
GAPDH
G6PD
GAPDH
AGS
MGC803
HGC27
MKN45

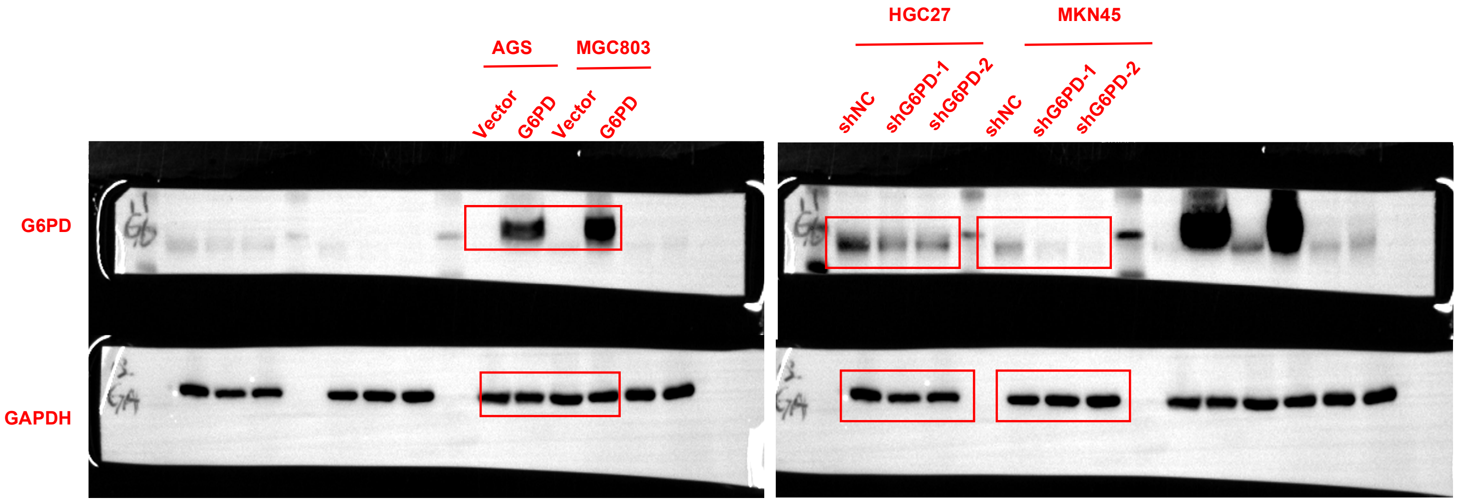
AGS
MGC803
HGC27
MKN45
Vector
G6PD
Vector
G6PD
shNC
shG6PD-1
shG6PD-2
shNC
shG6PD-1
shG6PD-2
G6PD
GAPDH
