# Supplementary material for: Histone modification-regulated LncRNA DLEU1 interacts with ASCC2/ALKBH3 complex to drive DNA repair, antioxidant homeostasis and glucose metabolism in gastric cancer
Source: Biomark Res. 2026 Jan 2;14:1. doi: 10.1186/s40364-025-00867-y (PMC12764130; doi:10.1186/s40364-025-00867-y)

**Histone modification-regulated LncRNA *DLEU1* interacts with ASCC2/ALKBH3 complex to drive DNA repair, antioxidant homeostasis and glucose metabolism in gastric cancer**

Xiaoyan Zhang^1,2,3#^, Xin Wang^1,2,3#^, Qi Wang^1,2,3#^, Xu Wang^1,2,3#^, Hui Sun^1,2,3^, Yingxue Liu^1,2,3^, Cong Tan^1,2,3^, Shujuan Ni^1,2,3^, Weiwei Weng^1,2,3^, Meng Zhang^1,2,3^, Lei Wang^1,2,3^, Dan Huang^1,2,3^, Jie Chen^2,4^, Xiaoyu Wang^5^, Lu Gan^6^, Mierxiati Abudurexiti^7^, Wenfeng Wang^2,8*^, Jinjia Chang^2,9*^, Weiqi Sheng^1,2,3*^, Midie Xu^1,2,3,10*^

**Supplementary Figures**

**Supplementary Figure 1. Effects of *DLEU1* overexpression and silencing on cellular behaviors in GC cells**

A. Relative expression levels of *DLEU1* in AGS and MGC803 cells following *DLEU1* overexpression, and in MKN45 and HGC27 cells upon *DLEU1* silencing.

B-E. Representative images and quantification of colony formation assay (B), wound healing assay (C), and transwell migration assay in AGS and MGC803 cells with *DLEU1* overexpression, and MKN45 and HGC27 cells with *DLEU1* silencing(D & E). Scale bar =200μm.

F. Comet assay showed DNA damage accumulation in HGC27 and MKN45 cells upon *DLEU1* Knockdown. Scale bar =20μm.

G. Western blot analysis of γH2AX expression in AGSand MGC803 cells with *DLEU1* overexpression, after treated with Etoposide (10 µM for 1 hour).

H. Flow cytometry analysis of apoptosis in AGS and MGC803 cells with *DLEU1* overexpression, and in MKN45 and HGC27 cells with *DLEU1* knockdown.

I. The mRNA expression levels of MMR genes (MLH1, MSH2, MSH6, PMS2) in AGS and MGC803 cells transfected with Vector or a *DLEU1*-overexpressing plasmid.

*, P < 0.05; **, P < 0.01; ***, P < 0.001; ns, not significant.


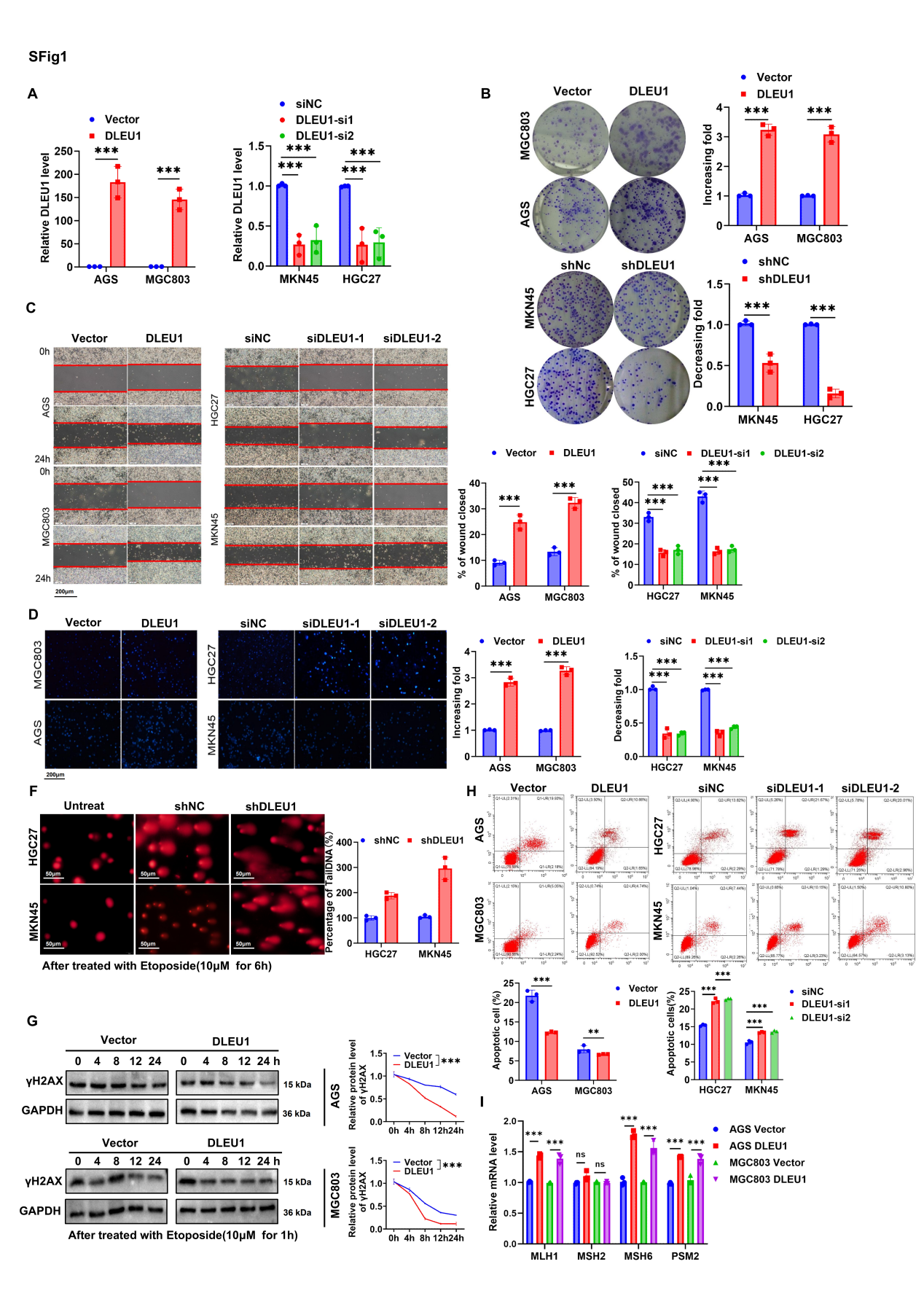


**Supplementary Figure 2. *DLEU1* modulates glucose metabolism and its knockdown suppresses tumor growth *in vivo***

A. Relative levels of glucose production, lactate secretion,, and ATP generation in AGS, MGC803, and HG227 cells following *DLEU1* overexpression or knockdown.

B. Seahorse extracellular flux analysis assessing the oxygen consumption rate (OCR) and extracellular acidification rate (ECAR) in gastric cancer (GC) cells with *DLEU1* overexpression.

C. The mRNA levels of metabolic genes (GLUT1, GLUT4, HK2, LDHA) in AGS, MGC803, and HG227 cells with *DLEU1* overexpression

D. Characterization of gastric cancer organoids derived from tumor tissues. Hematoxylin and eosin (HE) staining, as well as IHC analysis of CEA and CK7, were performed on both tumor tissues and corresponding organoids. Scale bar = 200 μm.

*, P < 0.05; **, P < 0.01; ***, P < 0.001; ns, not significant.


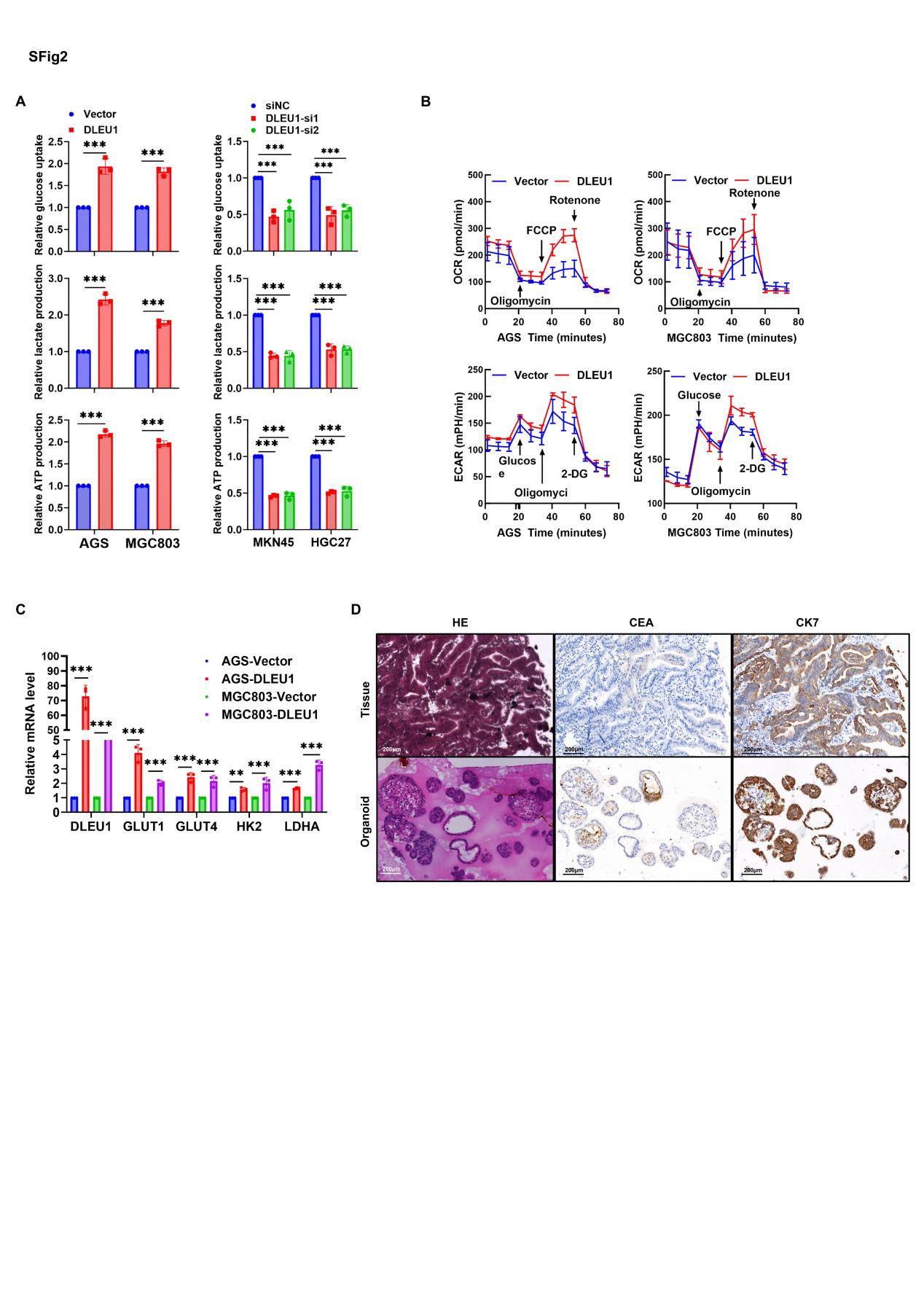


### **Supplementary Figure 3. Effects of *DLEU1*on ASCC2 expression**

A. Relative expression levels of *DLEU1* in AGS, MGC803, HGC27, and MKN45 cells after transfection with siNC or si*DLEU1* (si*DLEU1*-1 and si*DLEU1*-2).

B. Western blot analysis of ASCC2 and GAPDH in AGS and HGC27 cells after treatment with CHX at various time points (0, 3, 6, 9, 12, 24 hours). *DLEU1* overexpression (AGS) and *DLEU1* silencing (HGC27) were assessed.

*, P < 0.05; **, P < 0.01; ***, P < 0.001; ns, not significant


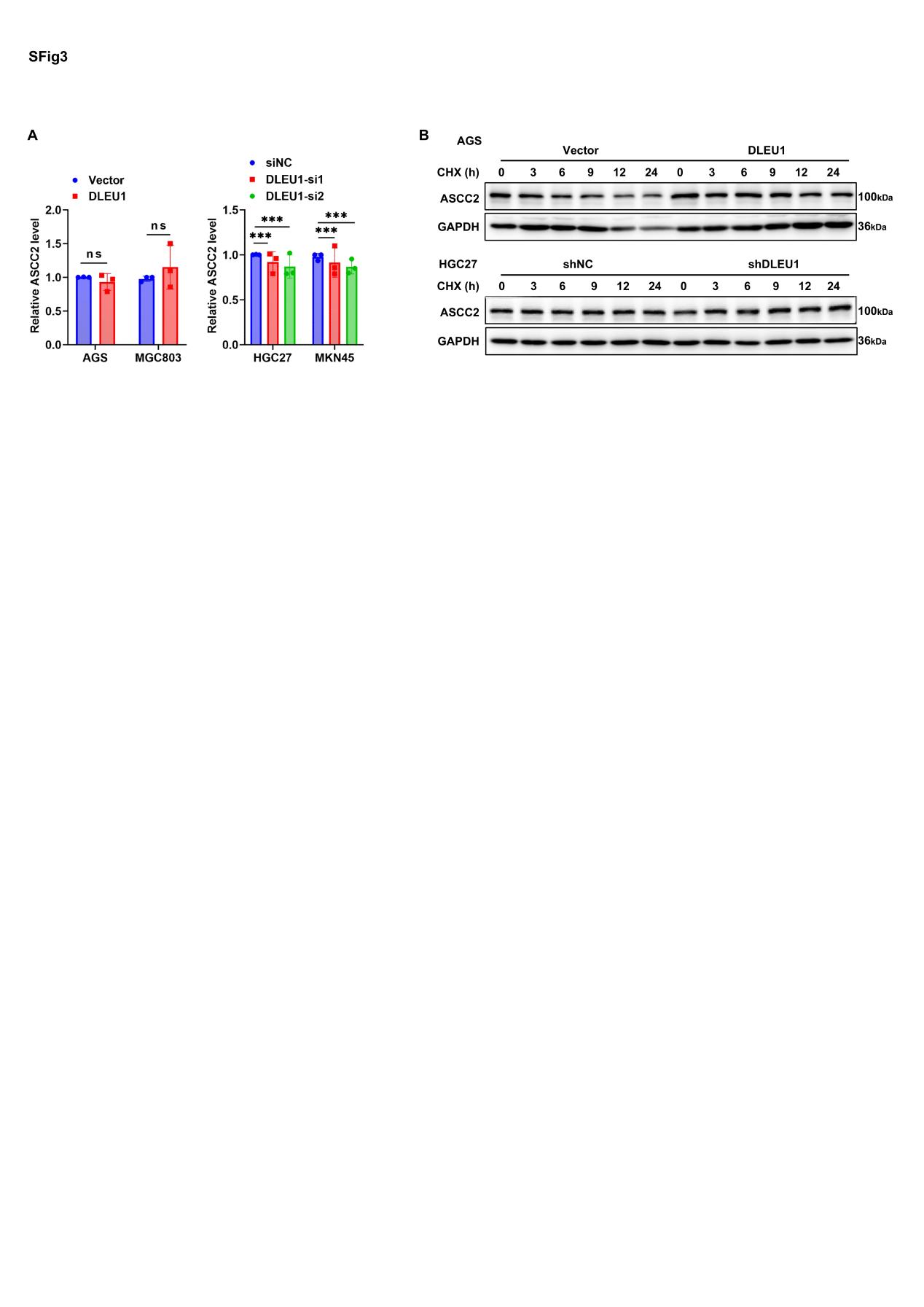
.

### **Supplementary Figure 4. Effects of G6PD on GC cells proliferation**

A-B. Western blot analysis of G6PD expression in AGS and MGC803 cells with G6PD overexpression, and in HGC27 and MKN45 cells with G6PD knockdown.

C. CCK8 assay showed cell proliferation in AGS and MGC803 cells with G6PD overexpression, and in HGC27 and MKN45 cells with G6PD knockdown.

D. Representative comet assay images in AGS and MGC803 cells with *DLEU1* overexpression combined with shG6PD, or G6PDi-1 treatment. Scale bar = 50 μm.

*, P < 0.05; **, P < 0.01; ***, P < 0.001; ns, not significant.


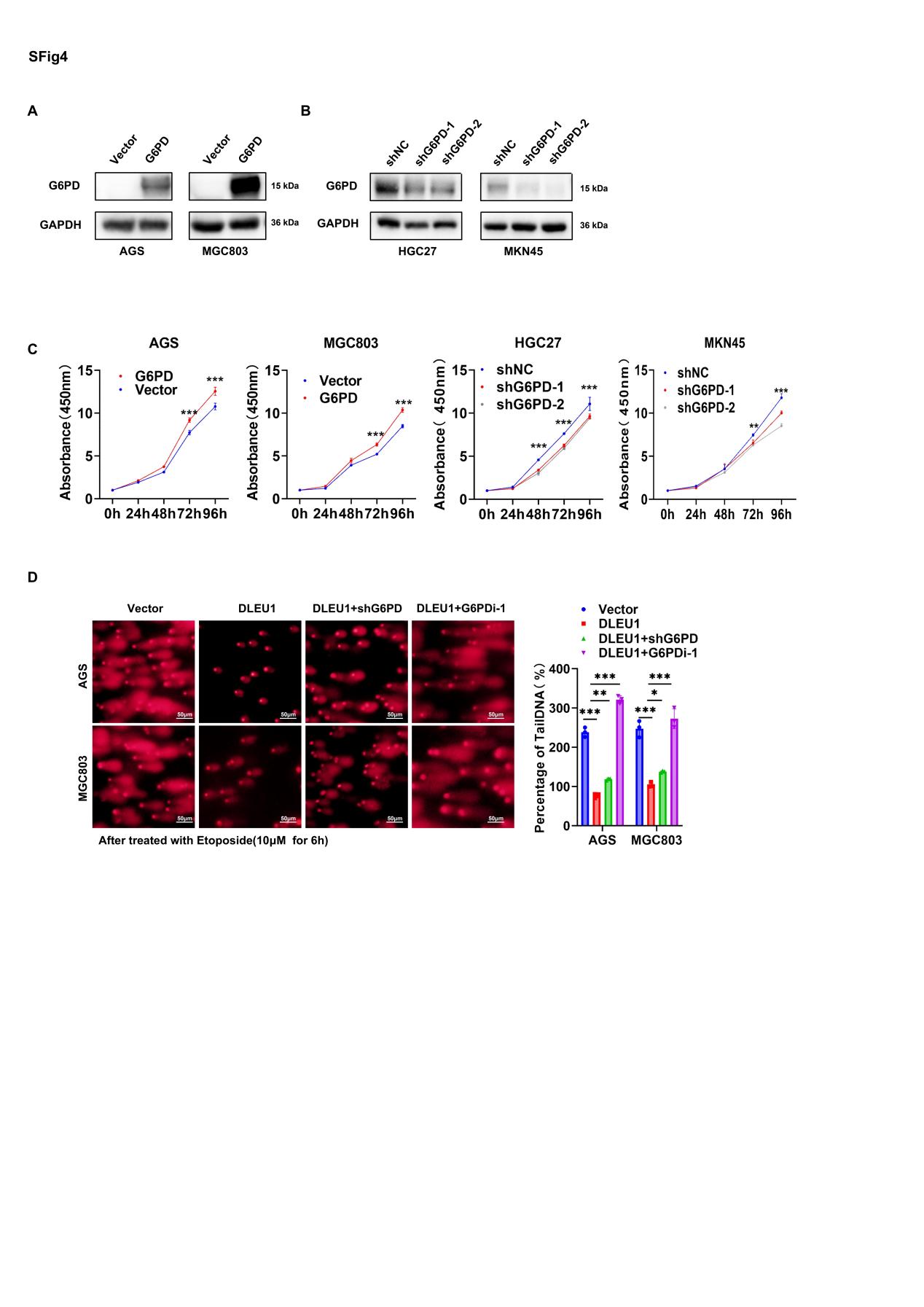

Supplement: Supplementary file 3 — Supplementary Material 3 [file 40364_2025_867_MOESM3_ESM.docx]
